# Optical fibre based artificial compound eyes for direct static imaging and ultrafast motion detection

**DOI:** 10.1038/s41377-024-01580-5

**Published:** 2024-09-18

**Authors:** Heng Jiang, Chi Chung Tsoi, Weixing Yu, Mengchao Ma, Mingjie Li, Zuankai Wang, Xuming Zhang

**Affiliations:** 1 Department of Applied Physics, The Hong Kong Polytechnic University, 999077 Hong Kong, China; 2 Photonics Research Institute (PRI), The Hong Kong Polytechnic University, 999077 Hong Kong, China; 3 Key Laboratory of Spectral Imaging Technology, Xi’an Institute of Optics and Precision Mechanics, Chinese Academy of Sciences, 710119 Xi’an, China; 4 Anhui Province Key Laboratory of Measuring Theory and Precision Instrument, School of Instrument Science and Opto-Electronics Engineering, Hefei University of Technology, 230009 Hefei, China; 5 Department of Mechanical Engineering, The Hong Kong Polytechnic University, 999077 Hong Kong, China; 6 Research Institute for Advanced Manufacturing (RIAM), The Hong Kong Polytechnic University, 999077 Hong Kong, China

**Keywords:** Imaging and sensing, Fibre optics and optical communications, Integrated optics, Optical sensors, Photonic devices

## Abstract

Natural selection has driven arthropods to evolve fantastic natural compound eyes (NCEs) with a unique anatomical structure, providing a promising blueprint for artificial compound eyes (ACEs) to achieve static and dynamic perceptions in complex environments. Specifically, each NCE utilises an array of ommatidia, the imaging units, distributed on a curved surface to enable abundant merits. This has inspired the development of many ACEs using various microlens arrays, but the reported ACEs have limited performances in static imaging and motion detection. Particularly, it is challenging to mimic the apposition modality to effectively transmit light rays collected by many microlenses on a curved surface to a flat imaging sensor chip while preserving their spatial relationships without interference. In this study, we integrate 271 lensed polymer optical fibres into a dome-like structure to faithfully mimic the structure of NCE. Our ACE has several parameters comparable to the NCEs: 271 ommatidia versus 272 for bark beetles, and 180^o^ field of view (FOV) versus 150–180^o^ FOV for most arthropods. In addition, our ACE outperforms the typical NCEs by ~100 times in dynamic response: 31.3 kHz versus 205 Hz for *Glossina morsitans*. Compared with other reported ACEs, our ACE enables real-time, 180^o^ panoramic direct imaging and depth estimation within its nearly infinite depth of field. Moreover, our ACE can respond to an angular motion up to 5.6×10^6^ deg/s with the ability to identify translation and rotation, making it suitable for applications to capture high-speed objects, such as surveillance, unmanned aerial/ground vehicles, and virtual reality.

## Abstract (web summary)

This artificial compound eye with microlensed optical fibres faithfully mimics the anatomical structure of natural compound eyes and thus can achieve 180^o^ superior static and dynamic perception.

## Graphical abstract

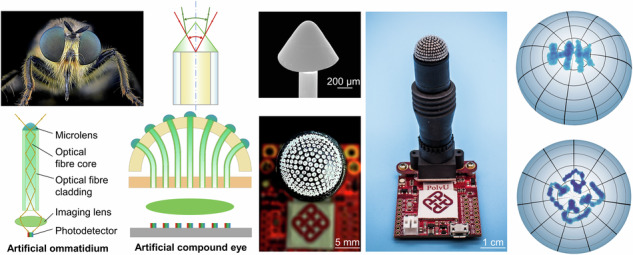

## Introduction

**Fig. 1:**
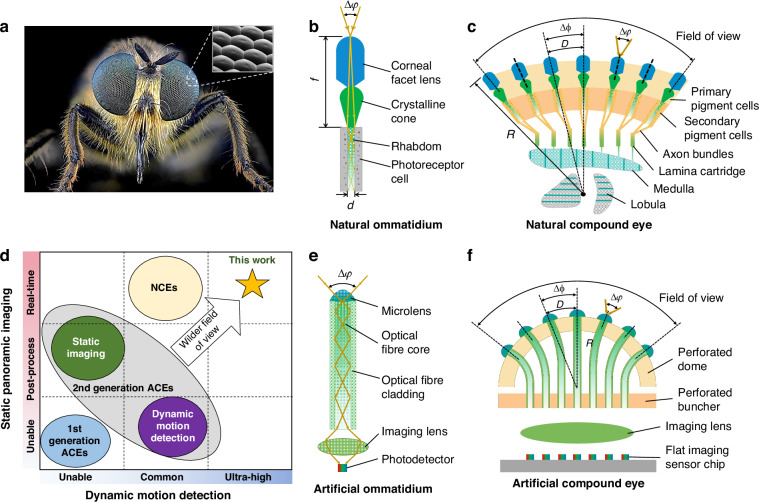
Concept and principle of the artificial compound eye for a panoramic camera (ACEcam) that uses conical-microlens optical fibres to mimic natural ommatidia. **a** The fly *Choerades fimbriata* has natural compound eyes (NCEs) for imaging; photograph courtesy of Mr. Thorben Danke of Sagaoptics. The inset shows compactly arranged corneal facet lenses in the NCEs. **b** In a natural ommatidium, the facet lens with a focal length *f* collects light at a specific acceptance angle Δ*φ*, the crystalline cone ensures light convergence, the rhabdom (diameter *d*) transmits light through the inner structure, and the photoreceptor cell records the light information. **c** An NCE consists of numerous natural ommatidia, which are surrounded by pigment cells to prevent crosstalk. Here, the interommatidial angle ∆Φ = *D*/*R*, where *D* and *R* denote the arc distance of adjacent ommatidia and the local radius of curvature, respectively. **d** Comparison of different compound eyes in the functions of static panoramic imaging and dynamic motion detection. The 1st generation ACEs primarily focused on the fabrication of ACE microlenses, lacking the ability of static imaging or dynamic detection. In the 2nd generation ACEs, none of these ACEs could realise real-time panoramic direct imaging and dynamic motion detection simultaneously, as what the NCEs can do. In contrast, our ACEcam is comparable to the NCEs in aspects of 180^o^ field of view and static imaging, and surpasses the NCEs in ultrafast motion detection. **e** An artificial ommatidium closely resembles a natural ommatidium by using a microlens to mimic the facet lens and the crystalline cone, an optical fibre core to mimic the rhabdom, an optical fibre cladding to mimic the pigment cells, an imaging lens to mimic synaptic units to focus each optical fibre onto an individual photodetector, and a photodetector in the flat imaging sensor chip to mimic the photoreceptor cell. **f** An artificial compound eye consists of numerous artificial ommatidia, with a flat imaging sensor chip mimicking the deeper neural centres (medulla and lobula), where signals are pre-processed. The signals are then transmitted to a computer for further analysis

Natural compound eyes (NCEs) were first investigated by Robert Hooke in 1665 after he observed orderly arranged *pearls* in the cornea of a grey drone fly (Fig. 1a)^1^. This research increased interest in NCEs^2^. Later, Sigmund Exner proposed the ommatidium as the basic unit of a compound eye. In each ommatidium of the NCE, light is first collected by a corneal facet lens (i.e., a *pearl*) at a certain acceptance angle and then transmitted by a crystalline cone and rhabdom (i.e., a light guide) to photoreceptor cells^3^ (Fig. 1b). Ommatidia are further innervated by axon bundles that execute synaptic connections in lamina cartridges^4^. After the primary signal is processed in deeper neural centres, such as the medulla and lobula (Fig. 1c)^5^, information is finally transmitted to central brain regions. Unlike the monocular eyes of vertebrates, NCEs utilise ommatidia arrayed on a curved surface (Fig. 1c). Furthermore, NCEs have many advantages, such as a panoramic field of view (FOV), good depth perception, negligible aberration, and fast motion tracking capability^6–8^.

NCEs have inspired the development of artificial compound eyes (ACEs) based on planar microlens arrays^9^, curved microlens arrays^10–14^, and metasurfaces^15^. In ACEs, the photodetector cells are arranged on either curved^11–13^ or planar^16^ surfaces. Nevertheless, most of the reported ACEs do not faithfully replicate the NCE structures and therefore lack some of their advantages. Since ACE designs using planar microlens arrays or metasurfaces usually have limited FOVs^17^, they are therefore not investigated in this study. ACEs with curved microlens arrays are briefly compared in Table 1 and elaborated in Supplementary Table S1. Specifically, the 1st generation ACEs primarily focused on the fabrication of ACE microlenses, lacking the ability to achieve panoramic imaging and dynamic detection^14,18^ (Fig. 1d). In the 2nd generation ACEs, some showed the capability of panoramic imaging, but they needed post-processing to retrieve the images, such as single-pixel imaging technique^19^, scanning method with mapping algorithm^12,13^, and backpropagation neural networks^20^. Despite recent efforts on real-time direct imaging, those ACEs still struggled with quantitative distance estimation^16^. Some other ACEs could realise dynamic motion detection but at common speeds^11^. Nevertheless, none of those ACEs can match the NCEs in achieving real-time panoramic direct imaging and dynamic motion detection simultaneously (Fig. 1d, Table 1, and Supplementary Table S1). Currently, the main challenge with curved microlens array-based ACEs is how to transmit the light rays collected by many microlenses on a curved surface to a flat imaging sensor (e.g., a CMOS chip) while maintaining their spatial relationships. Optical waveguides could address this challenge, as presented in one recent study^16^ that filled silicone elastomer into the hollow pipelines in a 3D-printed black substrate. Nevertheless, the waveguiding effect was unmet due to the layered texture and the black colour in the 3D-printed pipeline inner surfaces. Additionally, it did not mention the optical design criteria that are essential for optical waveguides in ACEs.

**Table 1:** Comparison of our ACEcam with reported artificial compound eyes (ACEs) and natural compound eyes (NCEs)

	1st generation ACEs		2nd generation ACEs				NCEs	Our ACEcam
Field of view	90^o^	140^o^	-	180^o^×60^o^	160^o^	170^o^	150^o^-180^o^	180^o^
Nearly infinite depth of field	√	√	√	√	√	×	√	√
Distance estimation	×	×	×	×	×	×	√	√
Optical guiding	×	×	√	×	×	√	√	√
Direct static imaging	×	×	×	×	×	√	√	√
Fast motion detection	×	×	×	×	×	×	√	Ultrahigh
Reference	^14^	^18^	^10^	^11^	^12^	^16^	^1,3,4,7,16,26^	-

Luckily, optical fibres, which majorly are divided into glass ones and plastic ones, are promising to solve this challenge since they can capture light at certain angles and transmit light over a long distance with very low loss^21^. They have been extensively applied in telecommunications^22^, sensors^23^, light guides^24^, and imaging systems^25^. Nevertheless, not a completed ACE system using optical fibres was once proposed to meet both 180^o^ real-time direct static imaging within the nearly infinite depth of field and dynamic perception with fast angular responses. This is because silica optical fibres are stiff and break easily at large bending angles, they are not suitable for ACEs^21^. Instead, the acceptance angle of plastic optical fibres is too high, lowering the angular resolution.

**Fig. 2:**
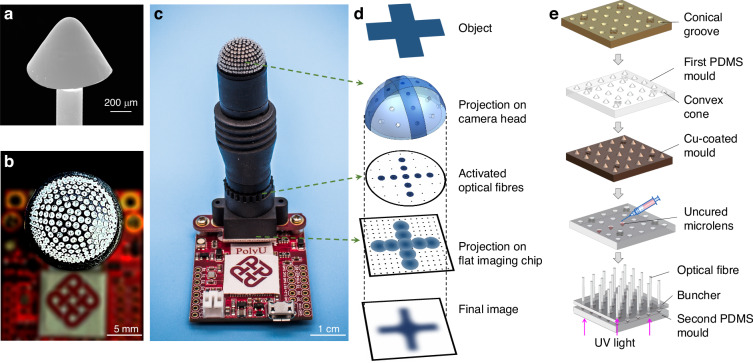
Operating principles and fabrication of the ACEcam. **a** Scanning electron microscopy (SEM) image of the conical microlens on an optical fibre. **b** Top view of the ACEcam light receiving head that uses a 3D-printed dome to host 271 fibre ends. **c** Photograph of an assembled ACEcam. **d** Concept of image formation. Using a ^‘^+^‘^ line-art pattern as the object (top panel), some fibres receive light from the object (second panel), and this pattern is transmitted from the lens end to the other end of the fibre (third panel). An imaging lens is employed to project the light from the fibre ends to a flat imaging sensor chip (fourth panel top), which is then converted into the final digital image (bottom panel). **e** Fabrication process flow of conical microlens optical fibres. A template with an array of conical grooves is fabricated by an ultrahigh precision 3D printing method (top panel), then the first PDMS mould is made to obtain convex cones (second panel). Physical vapour deposition and electroplating are then utilised to coat Cu layers on the first PDMS mould to smooth the rough surface of the convex cones and to round the sharp tip of the convex cones (third panel). After the second pattern transfer to get the second PDMS mould, optical adhesive NOA81 is dropped (0.15 μL/drop) into each conical groove by using a microsyringe (fourth panel). Next, an optical fibre buncher is mounted on the second PDMS mould so that optical fibres are well aligned with and submerged into the NOA81 microlenses wells. After UV illumination, each optical fibre end is mounted with a conical microlens, and finally, all fibres are peeled off (bottom panel)

In this work, we add a microlens to one end of a plastic optical fibre (Fig. 2a) to mimic the structure and functions of a natural ommatidium (Fig. 1e). The lensed ends of 271 fibres (versus 272 ommatidia for bark beetles^26^) are incorporated onto a curved surface (Figs. 1f, 2b) and used to assemble a biomimetic ACE as a panoramic camera (called ACEcam hereafter, Fig. 2c). The ACEcam faithfully mimics the structure of apposition ACEs and excels in both static and dynamic perceptions, thus finding niche applications in diverse imaging and dynamic detection domains.

## Results

### Assembled device

In the assembly, 271 lensed plastic optical fibres (Fig. 2a, details of fabrication will be explained below) are attached to a 3D-printed perforated dome (diameter 14 mm, Materials and methods: Fabrication of artificial compound eyes for a full-vision camera, Fig. 5a, b) so that all the lensed ends of the fibres are on the dome surface (Fig. 2b), while the bare ends of the fibres are placed into a perforated planar buncher (Fig. 5c). Light leaving the bare fibre ends is projected onto a flat imaging sensor via an imaging lens (Fig. 1f). The dome, the buncher, the imaging lens and a flat imaging sensor chip are hosted in a screwed hollow tube (Fig. 5d). In the assembly, the 3D-printed dome has the black colour so as to absorb the leaked or stray light, functioning the same as the pigment cells in the NCEs to prevent crosstalk. The lensed plastic fibres confine the collected light, preventing crosstalk and the associated ghost images, and the buncher maintains the relative positions of the microlenses on the dome. This setup enables the light collected at the curved surface to be transmitted to a flat image sensor, thus faithfully replicating the ommatidia in an NCE.

In the image formation process, the light emitted by the object is captured at different angles by the microlenses on the dome (Fig. 2d). At the bare fibre ends, the planar images are projected onto the flat imaging sensor chip. Then, the final images are obtained for digital image processing. The imaging lens prevents contact of the bare fibre ends with the vulnerable image chip surface.

In the NCE, if the ommatidium acceptance angle is ∆*φ* = *d*/*f* and the interommatidial angle is ∆Φ = *D*/*R* (Fig. 1b, c), ∆*φ* should be slightly larger than ∆Φ to ensure that no angular information is lost while reducing redundant angular overlap; here, *d*, *f*, *D* and *R* denote the rhabdom diameter, the focal length of the facet lens, the arc distance of adjacent ommatidia and the local radius of curvature, respectively^27^. Similarly, in ACEcam, ∆*φ* should be only slightly larger than ∆Φ = 12.2^o^ (Fig. 1f). Otherwise, the receiving areas of adjacent fibres would have a large overlap, lowering the angular resolution (Supplementary Fig. S1a).

Although plastic optical fibres are a good choice due to their flexibility and durability, the light acceptance angle of a plastic optical fibre is usually large (e.g., 60^o^, Materials and methods: Acceptance angle of the bare plastic optical fibre, Fig. 6a, b). Therefore, the acceptance angle of the optical fibre should be reduced by properly engineering the fibre tip (Supplementary Fig. S1b). Here, we add a microlens with a conical shape onto the distal end of the plastic optical fibre (Fig. 2a). Although spherical microlenses are easy to fabricate via surface tension and are thus widely used, the use of these microlenses often increases the acceptance angle (Materials and methods: Acceptance angle of the plastic optical fibre with a spherical microlens, Figs. 6c, d, 7a). In contrast, a conical microlens could reduce the acceptance angle (Fig. 7b); however, these microlenses with optical smoothness are more difficult to fabricate due to the unique shape and low melting point of plastic optical fibres. Our analysis and simulations show that a half-apex angle of *θ* = 35^o^ is the best choice for the conical microlens, reducing the acceptance angle of the fibre from 60^o^ to 45^o^ (Materials and methods: Acceptance angle of the plastic optical fibre capped with a conical microlens - Choice of shape and size of microlenses, Fig. 6e–h). Moreover, the sharp tip of the conical microlens is rounded during the fabrication process. This rounded tip is beneficial since it ensures that light information in the central angular range is not lost (Materials and methods: Choice of shape and size of microlenses 6, Fig. 8).

The conical microlens plastic optical fibres are fabricated in batches by a sequence of 3D printing, electroplating and two moulding processes (Fig. 2e, Materials and methods: Fabrication of a conical microlens on an optical fibre and Fig. 9), which is a novel approach to add a microlens onto the distal end of an optical fibre. Approximately 200 conical-microlens optical fibres are obtained in each batch process, and each conical microlens has a smooth surface and naturally a rounded tip (Fig. 2a). After the assembly process, the fabricated ACEcam is ready for experiments.

### Static imaging

**Fig. 3:**
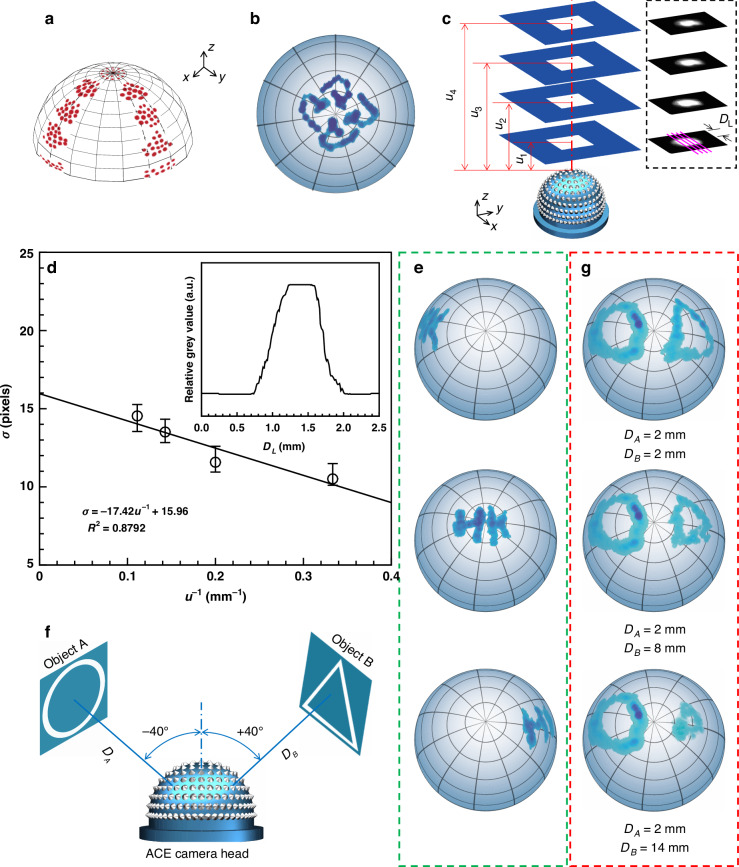
Static imaging and depth estimation of the ACEcam. **a** Combined image of a laser spot from nine angles (from −90^o^ to 90^o^ in both the *x* and *y* directions at a step of 22.5^o^). **b** Image of the logo of The Hong Kong Polytechnic University. **c**, **d** Depth estimation using the linear relationship between the point spread parameter *σ* and the reciprocal of the object distance *u*^−1^. In (**c**), example images at four different distances *u*_1_ = 3 mm, *u*_2_ = 5 mm, *u*_2_ = 7 mm, and *u*_4_ = 9 mm are shown in the dotted box. In the image acquired at each distance, the grey values along four parallel lines (shown here in pink) in the *x* direction are analysed to calculate the mean value and the errors shown in (**d**). *D*_*L*_ is the distance between a point on the pink line and the upper boundary of an image. In (**d**), the inset shows the relative grey value distribution along one sample pink line in **c**. A low error range signifies a high reliability of ACEcam^’^s depth estimation. **e**, Images of the letters ‘HK’ captured at three different polar angles relative to the centre of the camera: −50^o^ (top), 0^o^ (centre) and 50^o^ (bottom). **f** Schematic of an experimental setup to verify the nearly infinite depth of field of ACEcam. Objects A (circle) and B (triangle) are placed at angular positions of −40^o^ and 40^o^. **g** Images of the circle and triangle patterns when the distance of the circle image is fixed at *D*_*A*_ = 2 mm and the distance of the triangle image varies from *D*_*B*_ = 2, 8 to 14 mm

Previous studies on ACEs focused on static imaging (e.g., point-source tracking and panoramic imaging^12,13^) or dynamic motion extraction^11^. Nevertheless, static imaging usually requires a complex scanning system, which considerably reduces the imaging rate^12^, whereas dynamic motion extraction often obtains mosaic results due to the discrete distribution of photodetectors on the curved surface^11^. The proposed ACEcam can perform both static imaging and dynamic motion detection and has several advantages. First, this design has an exceptionally wide FOV (i.e., 180^o^). For experimental verification, laser spots are illuminated from 90^o^ to 0^o^ at steps of 22.5^o^ in both the *x* and *y* directions (see Fig. 3a for the combined result and Supplementary Fig. S2 for the individual images). Over the whole 180^o^ FOV, the images are highly uniform in size, brightness and angular position. This 180^o^ FOV helps ACEcam to exceed most ACEs to capture wider light information, and thus to be more suitable in various applications such as surveillance and unmanned drones.

Secondly, ACEcam also supports real-time panoramic direct imaging without distortions. Different test patterns, such as the logo of our university and the letters ‘HK’, can be imaged clearly with ACEcam (Fig. 3b, e and Supplementary Fig. S3). Unlike the ACEs developed in prior studies, which required redundant postprocessing approaches^12,13,19,20^, ACEcam enables direct imaging, similar to the capabilities of real NCEs. In addition, object distances can be estimated (i.e., depth estimation). In these experiments, a checkerboard pattern is set at different object distances from the camera (red lines in Fig. 3c), and the grey values at different distances along the vertical edge direction (pink lines in Fig. 3c) are analysed to determine the relationship between the point spread parameter *σ* and the reciprocal of the object distance *u*^−1^ (Fig. 3d), which should theoretically follow a linear relationship^28,29^ (Supplementary Section 2). Given the point spread parameter *σ* based on a measured image, the distance *u* can be obtained directly from the fitting expression. The absolute value of this slope is defined as the critical parameter *m* of the camera in this work, representing how the imaging quality of the camera is affected by the object distance. Here, the ACEcam is determined to have a value of *m* = 17.42 (Fig. 3d, Supplementary Section 2). If other images with unknown distances are captured, their *σ* values can be calculated, and then their distances *u* can be determined using the linear curve between *σ* and *u*^−1^. Next, the letters ‘HK’ are placed at three different angular positions: −50^o^ (left), 0^o^ (centre) and 50^o^ (right). No image distortions are observed (Fig. 3e), showing the good panoramic imaging performance of ACEcam. In comparison with other ACEs that attain panoramic imaging through redundant post-processing methods^12,13,19,20^, the capabilities of real-time direct imaging and object distance estimation of our ACEcam make it versatile across a broader range of applications. For instance, it can capture images and measure distances among moving objects in reality.

The third merit is the nearly infinite depth of field. To verify this property, two objects, a circle and a triangle, are placed at two widely separated angles and different distances (Fig. 3f). When the distances of both objects are the same, the image sizes are similar (Fig. 3g and Supplementary Video 1). When the circle image is kept static and the triangle image is moved away from ACEcam, the circle image size remains unchanged, but the triangle image size decreases. The focus is always retained. The nearly infinite depth of field of ACEcam is attributed to the image formation process, as each fibre captures all the light information within its acceptance angle, regardless of the object distance. Compared with the ACEs without infinite depth of field^16^, this characteristic enables the ACEcam to perform better in certain fields, including applications in virtual reality and augmented reality, contributing to an enhanced sense of realism in augmented reality experiences.

### Dynamic detection

**Fig. 4:**
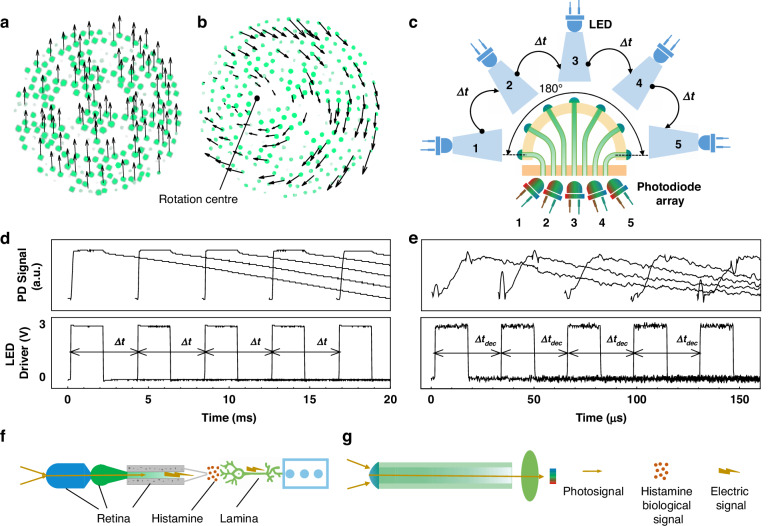
Dynamic motion detection of the ACEcam. **a** Optical flow as the ACEcam is translated in front of a checkerboard pattern at a distance of 10 mm. Here green dots represent the ommatidia illuminated by bright squares of the checkerboard, and the direction and length of the vector denote the motion direction and velocity of a bright square. **b** Optical flow as the ACEcam is rotated, with the dark spot indicating the calculated rotation centre. **c** Experimental setup to generate very high angular velocities for the dynamic response measurement. Five LEDs are evenly spaced along 180^o^ and lit up successively with a delay time ∆*t* whose minimum value is equal to the response time of the photodiode Δ*t*_*dec*_, and five photodiodes are employed to record the light emitted by the corresponding LEDs. **d**, **e** Response signals of the photodiodes (upper panel) when the LEDs are driven by square waves (lower panel) of *f*_*flicker*_ = 240 Hz in (**d**) and 31.3 kHz in (**e**). **f** Signal transmission pathway in the natural ommatidium. **g** Signal transmission pathway in the artificial ommatidium

Real-time perception ensures that the ACEcam is suitable not only for static imaging but also for dynamic detection. The fourth merit is that ACEcam can also be applied to determine optical flow according to visual translation and rotation signals. Here, the Lucas-Kanade method is adopted as the data processing algorithm due to its high efficiency in computing two-dimensional optical flow vectors based on images^30,31^ (Supplementary Section 3). In these experiments, when the ACEcam is placed 10 mm in front of a checkerboard pattern and moved vertically (Supplementary Fig. S4a), the computed optical flow vectors have uniform direction and length, illustrating the reliability and stability of ACEcam in dynamic motion detection (Fig. 4a). Since the checkerboard pattern has alternating dark and bright regions, the direction and length of the vector represent the direction and velocity of the motion of a bright region. Moreover, when the ACEcam is rotated (Supplementary Fig. S4b), the rotation centre can be easily identified (dark dot in Fig. 4b) since the length of the optical flow vector has a linear relationship with the distance from the rotation centre. This motion detection capability of ACEcam may facilitate various applications, such as kinestate tracking and motion state control in robots and unmanned aerial vehicles.

As the fifth merit, ACEcam shows ultrafast angular motion perception capability. To demonstrate a simple object with a fast angular motion, 5 LEDs are equally spaced over 180^o^ and sequentially activated by square waves with a period of Δ*t* (Fig. 4c). When a CMOS chip (OV7725, OmniVision Technologies Inc., 30 fps) is used as the photodetection unit, the frame rate is 30 Hz, and the angular perception is limited to 5.4 × 10^3^ deg/s (Materials and methods: Using a CMOS chip as the photodetection unit and Supplementary Video 2). Similarly, three ‘T’ objects are equally spaced over 180^o^ and sequentially activated by square waves with a period of Δ*t* (Supplementary Fig. S5) to further demonstrate the angular motion perception capability (Supplementary Video 3). Due to the frame rate limitations of the chip, a high flicker frequency *f*_*flicker*_ may lead to missing the recording of some objects. To further investigate ACEcam’s angular motion perception capability, a photodiode array with 5 electromagnetically shielded photodiodes (ElecFans) is used (Fig. 4c), and the light produced by flickering LEDs is recorded with these photodiodes. When *f*_*flicker*_ = 24 Hz is close to human flicker fusion frequency (FFF)^32,33^, the photodiodes get smooth response curves (Supplementary Fig. S6). And, when *f*_*flicker*_ = 240 Hz is close to the FFF of the fly *Glossina morsitans* (FFF = ~205 Hz^32,34^), the photodiode signals remain smooth (Fig. 4d). To test the limit, the LEDs are set at *f*_*flicker*_ = 31.3 kHz, which matches the response time of the photodiodes used in this experiment (i.e., 31.9 μs) and is ~100 times higher than the typical FFF of NCEs, the detected electrical signals of photodiodes change from a square wave to a spike wave (Fig. 4e). Equivalently, the ACEcam can respond to an angular velocity of up to 5.6 × 10^6^ deg/s (Materials and methods: Using a photodiode array as the photodetection unit and Supplementary Video 4), which can be further improved by several orders using faster photodiode arrays (e.g., 28 Gbit/s, PD20V4, Albis). This property broadens the application range to high-speed objects, such as aeroplanes and even spacecraft, a capability that is impossible for common ACEs.

The compelling reason behind ACEcam’s remarkable ultrafast angular motion perception lies in its emulation and surpassing of NCEs’ signal transmission. In contrast to spiking neurons, which exhibit an “all-or-none” behaviour due to their refractory period, nonspiking graded neurons in insects have multilevel responses and temporal summation characteristics when stimulated sequentially (Supplementary Fig. S7)^32,35^. This feature allows for a significant increase in the signal transmission rate between the retina and lamina neurons from approximately 300 bit/s (spiking neurons) to 1650 bit/s (nonspiking graded neurons)^36^. This feature enhances the performance of NCE visual systems. In our ACEcam, we have simplified the signal conversion process from that of natural ommatidium, which involves multiple steps (e.g., photosignals to histamine biological signals, then to electric signals; Fig. 4f); specifically, in the artificial ommatidium, only one signal transduction step is needed (i.e., photosignals to electric signals; Fig. 4g). Thus, the theoretical limit of the signal transmission rate is determined only by the frequency response of individual photodetection units (e.g., photodiodes), which could reach up to 50 Gbit/s, 7 orders of magnitude higher than that in the natural ommatidium. The distinctive anatomical structure empowers the ACEcam with significant potential for ultrafast angular motion perception, surpassing not only the existing ACEs but also outperforming the NCEs, and thus this characteristic serves as a blueprint for advancing ACE development.

## Discussion

In the proposed ACEcam, lensed plastic optical fibres are used as artificial ommatidia. By adding a conical microlens to the distal end of the fibre, the plastic optical fibre mimics the function of an ommatidium, collecting and transmitting light to the sensing unit. A bundle of lensed plastic optical fibres evenly distributed on a hemispherical surface is assembled to mimic NCEs, and the proposed ACEcam demonstrates excellent static imaging and dynamic motion detection capabilities. For example, a wide field of view (i.e., 180^o^) enables the ACEcam to outperform the majority of ACEs, making it particularly well-suited for applications in areas such as surveillance; the real-time panoramic direct imaging without distortions eliminates the need for redundant post-processing methods, rendering the ACEcam more suitable for applications such as imaging and distance measurement among moving objects in real-world scenarios; a nearly infinite depth of field can enhance the sense of realism in augmented reality experiences, making it more niche in virtual reality and augmented reality compared to those lacking this property; translational and rotational motion perception capabilities and ultrafast angular motion detection (5.6 × 10^6^ deg/s at maximum) provide the ACEcam with the potential for kinestate tracking and motion state control across various machines, from common cars to high-speed aeroplanes and even spacecraft. The amalgamation of these merits also positions the ACEcam for niche applications. For instance, the 180^o^ field of view and ultrafast angular motion detection make ACEcam suitable for integration into obstacle avoidance systems for high-speed unmanned aerial vehicles. This capability reduces the need for multiple obstacle avoidance lenses, consequently eliminating excess weight and size. The 180^o^ field of view and little size of ACEcam also let it suitable for endoscopy. Although the image resolution and size are limited by the number of artificial ommatidia, this ACEcam provides an overview of the imaging space, which is useful for complementing existing camera systems that observe regions of interest with high resolution to obtain fine details.

In future research, we plan to integrate apertures at the distal end of optical fibres. By considering the relationship among diameter, thickness, and distance of these apertures from the optical fibre, a further reduction in the acceptance angle could be anticipated. Besides, there are two approaches to improve the resolution and the miniaturisation of the ACE camera. (1) Narrower optical fibres: Plastic optical fibres with smaller diameters (e.g., from currently 250 μm to 25 μm) can be used to reduce the space occupied by each fibre and thus increase the number of plastic optical fibres. Additionally, advanced fabrication technologies and devices with better critical dimension capabilities (e.g., nanoArch S130, Boston Micro Fabrication Nano Material Technology) can be employed to create domes and bunchers with smaller dimensions and more through-holes with a reduced diameter. This would allow for a greater number of narrow plastic optical fibres, thereby enhancing the image resolution. Besides, the reduced diameters of key components would contribute to further miniaturisation of the ACEcam. (2) Optical fibre bundles: Plastic optical fibres can be replaced with imaging optical fibre bundles to mimic optical and neural superposition in NCEs. Since each imaging optical fibre bundle contains thousands of individual fibres, the resolution can be significantly increased if the relationship between the microlens and the imaging optical fibre bundles is well analysed, similar to the analysis presented in this article. More compact optical fibres in bundles will also aid in further miniaturising the ACEcam. Moreover, the combination of optofluidic lenses and ACEcam offers the potential to harness both benefits found in both arthropods’ compound eyes and vertebrate monocular eyes.

## Materials and methods

### Fabrication of artificial compound eyes for a full-vision camera

**Fig. 5:**
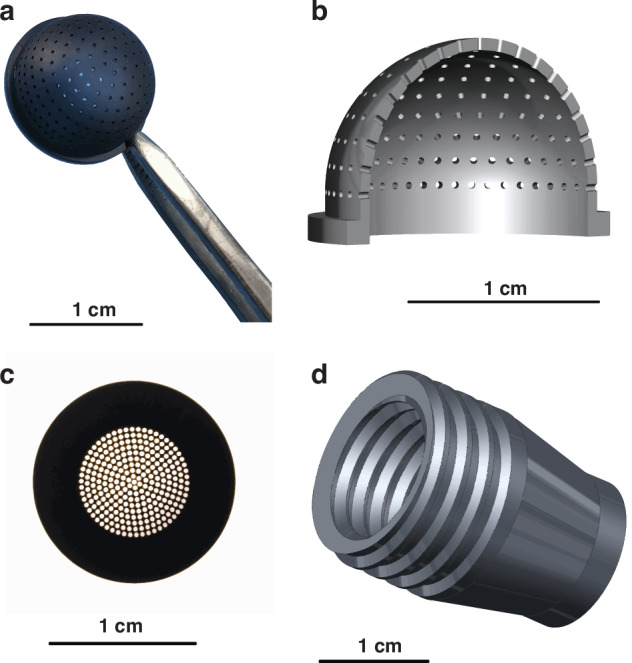
3D-printed components for the assembly of ACEcam. **a** Photograph of the perforated dome, held by tweezers. **b** Design of the perforated dome. **c** Photograph of the perforated buncher. **d** Design of the screwed hollow tube that will be used to hold the dome and the buncher

The 3D-printed components are illustrated in Fig. 5. First, a dome (Fig. 5a, b, external radius *R* = 7.0 mm, open angle 180^o^) and a buncher (Fig. 5c) were 3D-printed by projection micro stereolithography (microArch® S140, BMF Precision Tech Inc.). To allow for positioning each of the fibres, 271 through-holes with a diameter of 280 μm were evenly distributed in the dome (the number of through-holes from the centre to the outermost ring increases evenly, with values of 1, 6, 12, 18, 24, 30, 36, 42, 48, and 54, ensuring a uniform distribution) and another 271 through-holes were evenly distributed in the buncher. Then, 271 conical microlens optical fibres (external diameter *d* = 250 μm) were manually threaded through the holes in both the dome and the buncher while maintaining the relative positions of the optical fibres. The microlens ends of the optical fibres were placed on the curved surface of the dome, and the other ends of the optical fibres were cut to the same length after being passed through the buncher so that the fibre ends formed a flat surface. Next, the dome and the buncher were placed in a screwed hollow tube (Fig. 5d). This hollow tube was connected to another tube containing an imaging lens (standard M12 camera lens) and a flat imaging sensor chip (OV7725, OmniVision Technologies Inc.) (Figs. 1f, 2c). With this setup, the light rays received by the microlenses on the curved surface (i.e., the surface of the dome) could be transmitted to the planar surface (i.e., the surface of the buncher) and projected through the imaging lens to the flat surface of the imaging sensor chip.

### Acceptance angle of the bare plastic optical fibre

**Fig. 6:**
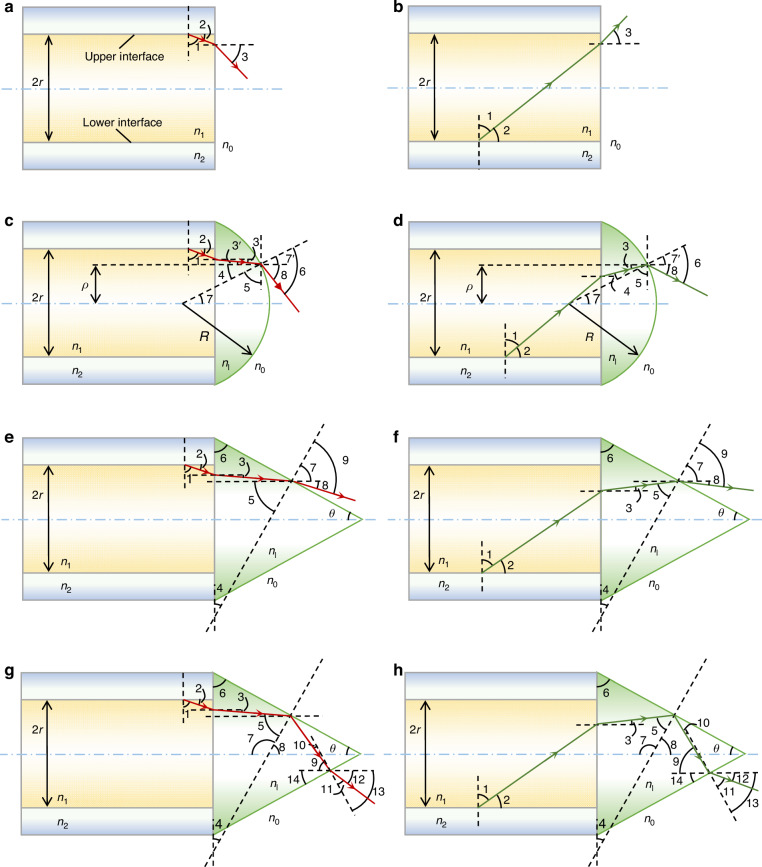
Light paths in different optical fibres for the analysis of the divergence angles (or equivalently, the acceptance angles). Red paths represent the light reflected from the upper core/cladding interface of the optical fibre, and green paths represent the light reflected from the lower core/cladding interface of the optical fibre. **a**, **b** In the bare multimode optical fibre, the light can be reflected from the upper (**a**) or lower (**b**) core/cladding interface. **c**,**d** In the optical fibre capped with a spherical microlens, the light can be reflected from the upper (**c**) or lower (**d**) core/cladding interface. **e**–**h** In the optical fibre capped with a conical microlens, the light has various paths. In one case, the light experiences no reflection in the conical surface after being reflected from the upper (**e**) or lower (**f**) core/cladding interface. In the other case, the light experiences one hop (i.e., reflection) in the conical surface after being reflected from the upper (**g**) or lower (**h**) core/cladding interface

A plastic optical fibre usually has a core (polymethyl methacrylate, PMMA) and a cladding (poly tetra fluoroethylene, PTFE). Based on the principle of optical path reversibility, the acceptance angle of the optical fibre is equal to the divergence angle when the light exits the fibre end. Therefore, we can study the divergence angles of optical fibres in different scenarios (Fig. 6).

In the simplest case of a bare multimode optical fibre, the light is reflected from the interface of the core and the cladding (Fig. 6). Let *n*_0_, *n*_1_, and *n*_2_ represent the refractive index of the air, core and cladding, respectively, and *r* denotes the core radius.

Specifically, when ∠1 reaches its minimum (Fig. 6a, b), it follows that

1 $${n}_{1}\,\sin \angle 1={n}_{2}\,\sin \frac{\pi }{2}$$

Similarly, when ∠2 reaches its maximum, it follows that

2 $$\sin \angle 2=\,\cos \angle 1=\,\cos ({\sin }^{-1}\frac{{n}_{2}}{{n}_{1}})=\frac{\sqrt{{n}_{1}^{2}-{n}_{2}^{2}}}{{n}_{1}}$$

Based on the law of refraction, at this time, the divergence angle ∠3 reaches its maximum absolute value when

3 $$\sin \angle 3=\frac{{n}_{1}}{{n}_{0}}\,\sin \angle 2=\frac{{n}_{1}}{{n}_{0}}\frac{\sqrt{{n}_{1}^{2}-{n}_{2}^{2}}}{{n}_{1}}=\frac{\sqrt{{n}_{1}^{2}-{n}_{2}^{2}}}{{n}_{0}}$$

Here, ${n}_{0}\,\sin \angle 3$ is also called the numerical aperture (NA). Typically, a plastic optical fibre has an *n*_0_ value of 1, with$\sqrt{{n}_{1}^{2}-{n}_{2}^{2}}=0.5$, and thus, ∠3 = 30^o^.

Similarly, when light is reflected from the opposite side of the interface between the core and cladding (Fig. 6b), the maximum absolute value of ∠3 is 30^o^. Therefore, the acceptance angle *φ*_*flat*_ of the flat end of the plastic optical fibre is *φ*_*flat*_ = 60^o^.

### Acceptance angle of the plastic optical fibre with a spherical microlens

Here, we analyse the acceptance angle of the plastic optical fibre with a spherical or conical microlens (Materials and methods: Acceptance angle of the plastic optical fibre capped with a conical microlens) based on light paths. When the light is reflected from the upper core/cladding interface (Fig. 6c), it is first refracted at the fibre/microlens interface and then at the microlens/air interface. Let *ρ*, *R* and *n*_*l*_ represent the radial position at the microlens surface, the radius of the microlens surface and the refractive index of the microlens, respectively. Then, ∠3 and ∠5 can be calculated as follows:

4a $$\angle 3={\rm{sin}}^{-1}\frac{\sqrt{{n}_{1}^{2}-{n}_{2}^{2}}}{{n}_{l}}$$

4b $$\angle 5={\rm{cos}}^{-1}\frac{\rho }{R}$$

Since ∠5 and ∠4 are complementary, we have

5 $$\angle 4=\frac{\pi }{2}-\angle 5=\frac{\pi }{2}-{\cos }^{-1}\frac{\rho }{R}$$

Then,

6 $$\angle 3^{\prime} +\angle 4=\angle 3+\angle 4=\frac{\pi }{2}+{\sin }^{-1}\frac{\sqrt{{n}_{1}^{2}-{n}_{2}^{2}}}{{n}_{l}}-{\cos }^{-1}\frac{\rho }{R}$$

Based on the law of refraction, we have

7 $$\sin (\angle 7^{\prime} +\angle 8)={n}_{l}\,\sin (\angle 3^{\prime} +\angle 4)={n}_{l}\,\cos \left({\sin }^{-1}\frac{\sqrt{{n}_{1}^{2}-{n}_{2}^{2}}}{{n}_{l}}-{\cos }^{-1}\frac{\rho }{R}\right)$$

8 $$\angle 7^{\prime} =\angle 7={\sin }^{-1}\frac{\rho }{R}$$

Finally, we have that

9 $$\angle 8={\sin }^{-1}\left[{n}_{l}\,\cos \left({\sin }^{-1}\frac{\sqrt{{n}_{1}^{2}-{n}_{2}^{2}}}{{n}_{l}}-{\cos }^{-1}\frac{\rho }{R}\right)\right]-{\sin }^{-1}\frac{\rho }{R}$$

In addition, the angle of the tangent line (Supplementary Fig. S8) at the curved surface is defined as follows:

10 $${\rm{Angle}}\,{\rm{of}}\,{\rm{tangent}}\,{\rm{line}}={\cos }^{-1}\left(\frac{\rho }{R}\right)$$

Thus, the ***upper*** limit angle *α*_*upper*_ should be the smaller value between ∠8 and the angle of the tangent line. The analytic results are plotted in Supplementary Fig. S9a for microlenses with different radii.

On the other hand, when light is reflected from the lower core/cladding interface (Fig. 6d), ∠3, ∠5 and ∠7 can be calculated with Eqs. (4a), (4b) and (8), respectively. However, ∠4 becomes

11 $$\angle 4=\frac{\pi }{2}-(\angle 3+\angle 5)$$

Based on the law of refraction, we have

12 $$\sin \angle 6={n}_{l}\,\sin \angle 4={n}_{l}\,\cos \left({\sin }^{-1}\frac{\sqrt{{n}_{1}^{2}-{n}_{2}^{2}}}{{n}_{l}}+{\cos }^{-1}\frac{\rho }{R}\right)$$

Since ∠6 = ∠7 + ∠8, ∠8 can be expressed as

13 $$\angle 8={\sin }^{-1}\left[{n}_{l}\,\cos \left({\sin }^{-1}\frac{\sqrt{{n}_{1}^{2}-{n}_{2}^{2}}}{{n}_{l}}+{\cos }^{-1}\frac{\rho }{R}\right)\right]-{\sin }^{-1}\frac{\rho }{R}$$

This gives the ***lower*** limit angle *α*_*lower*_ = ∠8. The analytic results are plotted in Supplementary Fig. S9b for microlenses with different radii.

Based on the analysis of the optical path, if *α*_*lower*_ ≤ 0, the half acceptance angle *φ*_*sm*_ should follow *φ*_*sm*_/2 = *α*_*upper*_; here, the subscript *sm* represents the spherical microlens. In contrast, if *α*_*lower*_ > 0, the half acceptance angle should follow *φ*_*sm*_/2 = *α*_*upper*_ - *α*_*lower*_. The analytic results are plotted in Supplementary Fig. S9c for microlenses with different radii, assuming that the material of the microlenses is NOA81 and the refractive index *n*_*l*_ is 1.56 (NOA81, a widely used UV-curable optical adhesive, meets both economic and optical practicality requirements, making it highly suitable as the material for microlenses). In this case, a hollow area is observed in the centre of the acceptance area. A detailed explanation of the cone will be presented below.

According to the theoretical analysis results (Supplementary Fig. S9c), we find that:

1. The acceptance angles *φ*_*sm*_ at different radial positions *ρ* vary significantly with the microlens radius *R*, increasing the difficulty of design and analysis.
2. In many cases, the acceptance angle has *φ*_*sm*_ > 60^o^, which is similar to the acceptance angle of 60^o^ of a bare plastic optical fibre. Thus, the use of a spherical microlens does not reduce the acceptance angle.

**Fig. 7:**
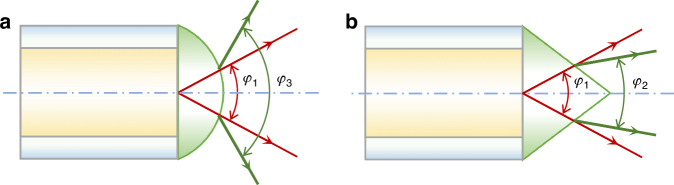
Comparison of the acceptance (divergence) angles of the plastic optical fibres with spherical or conical microlenses, with red paths representing the light emitted directly from the bare end of plastic optical fibres, and green paths representing the light emitted from microlenses. **a** The spherical microlens has a larger acceptance angle than the flat-end optical fibre (i.e., *φ*_3_ > *φ*_1_). **b** The conical microlens has a smaller acceptance angle (i.e., *φ*_2_ < *φ*_1_). Therefore, the use of conical microlenses can effectively narrow the acceptance angle

Therefore, the spherical microlens is not a good choice if the acceptance angle of the optical fibre needs to be reduced. Instead, we use a conical microlens to reduce the acceptance angle (Fig. 7).

### Acceptance angle of the plastic optical fibre capped with a conical microlens

The acceptance angle (or equivalently, the divergence angle) is strongly affected by whether the light experiences total internal reflection at the conical surface. Thus, we consider two cases, *no reflection* (Fig. 6e, f) and *one hop* (Fig. 6g, h). Here “hop” means “reflection” at the conical surface (not at the core/cladding interface). These two cases are discussed separately below.

#### No reflection at the conical surface of the microlens

When light is reflected from the upper core/cladding interface (Fig. 6e), ∠3 follows Eq. (4a). In addition, based on the geometrical relationship, ∠5 and ∠6 follow

14 $$\angle 5=\angle 6=\angle 7=\frac{\pi }{2}-\theta$$

where *θ* is half-apex angle of the cone.

Then, based on the law of refraction, we have

15 $$\begin{array}{l}\sin \angle 9={n}_{l}\,\sin (\angle 3+\angle 5)={n}_{l}\,\sin \left({\sin }^{-1}\frac{\sqrt{{n}_{1}^{2}-{n}_{2}^{2}}}{{n}_{l}}+\frac{\pi }{2}-\theta \right)\\\qquad\quad \,={n}_{l}\,\cos \left({\sin }^{-1}\frac{\sqrt{{n}_{1}^{2}-{n}_{2}^{2}}}{{n}_{l}}-\theta \right)\end{array}$$

Since ∠9 = ∠7 + ∠8, ∠8 is defined as

16 $$\angle 8={\sin }^{-1}\left[{n}_{l}\,\cos \left({\sin }^{-1}\frac{\sqrt{{n}_{1}^{2}-{n}_{2}^{2}}}{{n}_{l}}-\theta \right)\right]-\frac{\pi }{2}+\theta$$

Similarly, the ***upper*** limit angle *α*_*upper*_ should be the smaller value of ∠8 and the angle of the tangent line, which is equal to *θ*. In *φ*_*cm*_, the subscript *cm* denotes the conical microlens.

When light is reflected from the lower core/cladding interface (Fig. 6f), ∠3 still follows Eq. (4a). In addition, based on the geometrical relationship, ∠4 follows

17 $$\angle 4=\theta$$

Therefore, ∠5 follows

18 $$\angle 5=\frac{\pi }{2}-\angle 3-\angle 4=\frac{\pi }{2}-{\sin }^{-1}\frac{\sqrt{{n}_{1}^{2}-{n}_{2}^{2}}}{{n}_{l}}-\theta$$

Based on the geometrical relationship, ∠7 follows

19 $$\angle 7=\angle 3+\angle 5=\frac{\pi }{2}-\theta$$

Then, according to the law of refraction, ∠9 follows

20 $$\begin{array}{l}\sin \angle 9={n}_{l}\,\sin \angle 5={n}_{l}\,\sin \left(\frac{\pi }{2}-{\sin }^{-1}\frac{\sqrt{{n}_{1}^{2}-{n}_{2}^{2}}}{{n}_{l}}-\theta \right)\\\qquad\quad={n}_{l}\,\cos \left({\sin }^{-1}\frac{\sqrt{{n}_{1}^{2}-{n}_{2}^{2}}}{{n}_{l}}+\theta \right)\end{array}$$

Since ∠9 = ∠7 + ∠8, ∠8 is defined as

21 $$\angle 8={\sin }^{-1}\left[{n}_{l}\,\cos \left({\sin }^{-1}\frac{\sqrt{{n}_{1}^{2}-{n}_{2}^{2}}}{{n}_{l}}+\theta \right)\right]-\frac{\pi }{2}+\theta$$

Similarly, the ***lower*** limit angle *α*_*lower*_ should be the smaller value of ∠8 and the angle of the tangent line, which is equal to *θ*. In addition, when *α*_*lower*_ ≤ 0, the half acceptance angle *φ*_*cm*_ (or equivalently, the half divergence angle) follows *φ*_*cm*_/2 = *α*_*upper*_ (Supplementary Fig. S10a), and when *α*_*lower*_ > 0, *φ*_*cm*_ follows *φ*_*cm*_/2 = *α*_*upper*_–*α*_*lower*_ (Supplementary Fig. S10b). Thus, we find that the acceptance angle of the cone is independent of the radial position *ρ* of the point at which the light hits the conical surface and is only dependent on the half-apex angle *θ*. This feature is very different from that of the spherical microlens and also makes it easy for analysis.

#### One hop at the conical surface of the microlens

When the light is reflected from the upper core/cladding interface of the optical fibre, ∠3 still follows Eq. (4a) (Fig. 6g). Based on the geometrical relationship, ∠5 and ∠6 can be expressed as

22 $$\angle 5=\frac{\pi }{2}-\theta +\angle 3$$

23 $$\angle 6=\frac{\pi }{2}-\theta$$

Since the sum of the angles in the quadrilateral is 2*π*, we can formulate the following relationship:

24 $$\angle 9+\angle 14=2\pi -\left(\frac{\pi }{2}-\angle 3\right)-\angle 6-2\angle 5=\frac{3\pi }{2}+\angle 3-\angle 6-2\angle 5$$

Next, because

25 $$\angle 10=\frac{\pi }{2}-(\angle 9+\angle 14)=\angle 6+2\angle 5-\angle 3-\pi$$

we have that

26 $$\begin{array}{l}\angle 10=\left(\frac{\pi }{2}-\theta \right)+2\left(\frac{\pi }{2}-\theta +\angle 3\right)-\angle 3-\pi =\frac{\pi }{2}-3\theta +\angle 3\\\qquad =\frac{\pi }{2}-3\theta +{\sin }^{-1}\frac{\sqrt{{n}_{1}^{2}-{n}_{2}^{2}}}{{n}_{l}}\end{array}$$

Based on the law of refraction, we have

27 $$\angle 11={\sin }^{-1}({n}_{l}\,\sin \angle 10)$$

In addition,

28 $$\angle 13=\angle 9+\angle 10=\angle 6$$

Therefore,

29 $$\angle 12=\angle 13-\angle 11=\angle 6-\angle 11=\frac{\pi }{2}-\theta -{\sin }^{-1}\left[{n}_{l}\,\sin \left(\frac{\pi }{2}-3\theta +{\sin }^{-1}\frac{\sqrt{{n}_{1}^{2}-{n}_{2}^{2}}}{{n}_{l}}\right)\right]$$

Moreover, the angle of the tangent line is –*θ*. Therefore, when ∠12 ≥ 0, the upper limit angle should be ∠12, and when ∠12 < 0, the upper limit angle should be the smaller one of the absolute values of ∠12 and the tangent angle (i.e., the minimum of abs(∠12) versus *θ*, here abs means taking the absolute value).

When the light is reflected from the lower core/cladding interface of the optical fibre (Fig. 6h), ∠3 and ∠6 follow Eqs. (4a) and (23). Then, based on the geometrical relationship, ∠5 is defined as

30 $$\angle 5=\frac{\pi }{2}-\theta -\angle 3$$

Based on the sum of the angles in the quadrilateral, we have

31 $$\angle 9+\angle 14=2\pi -\left(\frac{\pi }{2}+\angle 3\right)-\angle 6-2\angle 5=\frac{3\pi }{2}-\angle 3-\angle 6-2\angle 5$$

Then, we have

32 $$\begin{array}{l}\angle 10=\frac{\pi }{2}-(\angle 9+\angle 14)=\angle 3+\angle 6+2\angle 5-\pi \\\qquad=\angle 3+(\frac{\pi }{2}-\theta )+2(\frac{\pi }{2}-\theta -\angle 3)-\pi \\\qquad =\frac{\pi }{2}-3\theta -\angle 3=\frac{\pi }{2}-3\theta -{\sin }^{-1}\frac{\sqrt{{n}_{1}^{2}-{n}_{2}^{2}}}{{n}_{l}}\end{array}$$

Based on the law of refraction, we have

33 $$\angle 11={\sin }^{-1}({n}_{l}\,\sin \angle 10)$$

In addition, we have

34 $$\angle 13=\angle 9+\angle 10=\angle 6$$

Therefore, we find that

35 $$\angle 12=\angle 13-\angle 11=\angle 6-\angle 11=\frac{\pi}{2}-\theta -{\sin}^{-1}\left[{n}_{l}\,\sin \left(\frac{\pi}{2}-3\theta -{\sin }^{-1}\frac{\sqrt{{n}_{1}^{2}-{n}_{2}^{2}}}{{n}_{l}}\right)\right]$$

Therefore, when ∠12 ≥ 0, the lower limit angle should be ∠12, and when ∠12 < 0, the lower limit angle should be the smaller of the absolute values of ∠12 and the angle of the tangent line (i.e., the minimum of abs(∠12) versus *θ*).

#### Analysis of the acceptance angle

**Fig. 8:**
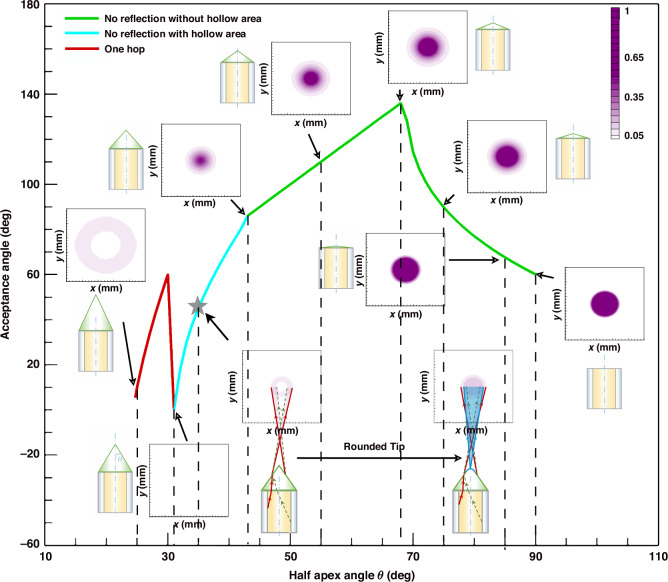
Acceptance angle as a function of the half-apex angles *θ* of the conical microlens. The theoretical analysis is presented using three distinct colour lines to illustrate different scenarios. (1) When *θ* ≥ 43^o^, the green line represents the case in which light is directly emitted from the conical surface of the microlens without any reflection, and there is no hollow region within the emission pattern. However, the acceptance angle is too large ( > 60^o^). (2) When 31^o^ ≤ *θ* < 43^o^, the cyan line represents the case in which light is directly emitted from the conical surface of the microlens without reflection. The acceptance angle is narrowed when *θ* goes smaller, but a hollow central region appears in the emission pattern. Equivalently, if the fibre collects light, the information in the central hollow region cannot be detected, which is unfavourable. The star highlights the working conditions used in our experiments, i.e., *θ* = 35^o^ and an acceptance angle of 45^o^. By rounding the sharp tip of the cone, the hollow central region can be eliminated from the emission pattern (the inset in the lower right part). (3) When *θ* < 31^o^, the red line represents the case in which the light undergoes a single reflection (or hop) in the conical microlens. The hollow central region reappears and the transmitted light intensity is very low. Therefore, this case is not suitable for collecting the light

Based on theoretical optical path analysis, when *θ* > 31^o^, the lights emitted by the optical fibres that first impact the conical surface are directly refracted (corresponding to the no-reflection case discussed above; Fig. 6e, f), while the one hop case can be ignored. In contrast, when *θ* < 31^o^, the lights that first impact the conical surface experience total internal reflection and thus are reflected once before going out (corresponding to the one hop case discussed above; Fig. 6g, h and the red line in Fig. 8). Nevertheless, the one hop case has low output energy. Therefore, we ignore the one hop case in the following discussions.

Supplementary Fig. S10a and Fig. S10b depict two scenarios of light transmission from the cone. When *α*_*lower*_ ≤ 0 (*θ* ≥ 43^o^), the critical light from the lower core/cladding interface travels upwards, and the light projected onto the receiving surface forms a circle on the observation screen. Since the microlens is axisymmetric, the actual acceptance area (or equivalently, the divergence area) has a circle shape with no hollow region (Supplementary Fig. S10a). Consequently, the acceptance angle of the cone is determined by the larger absolute value between *α*_*upper*_ and *α*_*lower*_. Notably, the absolute value of *α*_*upper*_ is consistently larger than *α*_*lower*_ based on the theoretical analysis. Therefore, the half acceptance angle *φ*_*cm*_/2 of the conical microlens is ultimately determined by *α*_*upper*_ (i.e., *φ*_*cm*_/2 = *α*_*upper*_) when *α*_*lower*_ ≤ 0 (Supplementary Fig. S10a, the green line segments in Fig. 8).

However, when *α*_*lower*_ > 0 (31^o^ < *θ* < 43^o^), the critical light from the lower core/cladding interface travels downwards, and the light projected onto the receiving surface forms a circle on the observation screen (Supplementary Fig. S10b). The emission pattern on the observation screen is a ring with a hollow region at the centre. In this case, the half acceptance angle of the cone is determined by the difference between both critical angles, that is, *φ*_*cm*_/2 = *α*_*upper*_ - *α*_*lower*_ (Supplementary Fig. S10b, the cyan line segment in Fig. 8). The hollow central region is unfavourable since light information in that angular range is lost.

To eliminate the hollow central region, the tip of the cone can be rounded (Supplementary Fig. S10c). With the rounded tip, the cone can project light to the central region. Equivalently, when used to receive light, the rounded tip can accept light from the central region.

### Optical tracing simulation

An optical tracing simulation is conducted to verify the designs of the cone. Under each condition, the light observation screen is placed 15 cm away from the cone, the cone bottom radius is 0.15 mm, the cone height is determined by *θ*, and the refractory index at 550 nm is 1.56. The cone sits directly on the flat end of an optical fibre with the following parameters: length, 10 mm; core material, PMMA; core diameter, 0.24 mm; core refractory index, 1.4936 at 550 nm; cladding material, PTFE; cladding diameter, 0.25 mm; and cladding refractory index, 1.4074 at 550 nm. These parameters are consistent with the optical fibres employed in this study. The light is introduced into the other end of the optical fibre and emitted from the cone, forming an emission pattern (i.e., a light intensity distribution) on the observation screen (Supplementary Fig. S10). As *θ* varies, the emission pattern changes considerably.

We consider the acceptance angle and the corresponding simulated optical field patterns for conical microlens optical fibres at different half-apex angles *θ* (Fig. 8). When *θ* ≤ 31^o^, no light is directly emitted from the cone. Hence, *θ* = 31^o^ is the minimum half-apex angle of the conical microlens. As *θ* decreases (*θ* < 31^o^), the light is reflected one or more times (hops) within the cone before being emitted, and the emission pattern is finally displayed (the red line segments in Fig. 8). When 31^o^ < *θ* < 43^o^, the acceptance angle increases with larger *θ*, and a hollow region appears in the middle of the emission pattern (the cyan line segment in Fig. 8). When 43^o^ ≤ *θ* ≤ 68^o^, the acceptance angle increases further with increasing *θ*, and the emission pattern is a solid circle (the straight part of the green line segment in Fig. 8). When *θ* > 68^o^, the acceptance angle decreases as *θ* increases, and the emission pattern is still a solid circle (the curved part of the green line segment in Fig. 8). The simulation results are quantitatively consistent with the theoretical results.

### Choice of shape and size of microlenses

In the ACEcam, the interommatidial angle ∆Φ is 12.2^o^, while the acceptance angle of the flat-end plastic optical fibre is 60^o^, causing severe overlap between the views of adjacent ommatidia (Supplementary Fig. S1a). Based on the above analyses, we choose a conical microlens with the half-apex angle *θ* = 35^o^ (marked with a star in Fig. 8). This value serves as a balance between the acceptance angle and the output energy. Specifically, when *θ* exceeds 35^o^, the acceptance angle becomes elevated; conversely, if *θ* falls below 35^o^, the resultant output energy diminishes to an impractical extent. Correspondingly, the acceptance angle is 45^o^. At *θ* = 35^o^, a hollow centre in the emission pattern cannot be avoided (Fig. 8). To address this issue, the sharp tip of the conical microlens is rounded. With this approach, the rays passing through the rounded part of the conical microlens are refracted towards the central part of the emission pattern, eliminating the hollow centre (Fig. 8). In summary, we choose a conical microlens with a rounded tip, a half-apex angle *θ* of 35^o^, and an acceptance angle is 45^o^ to ensure that the emission pattern has no hollow centre.

### Fabrication of a conical microlens on an optical fibre

**Fig. 9:**
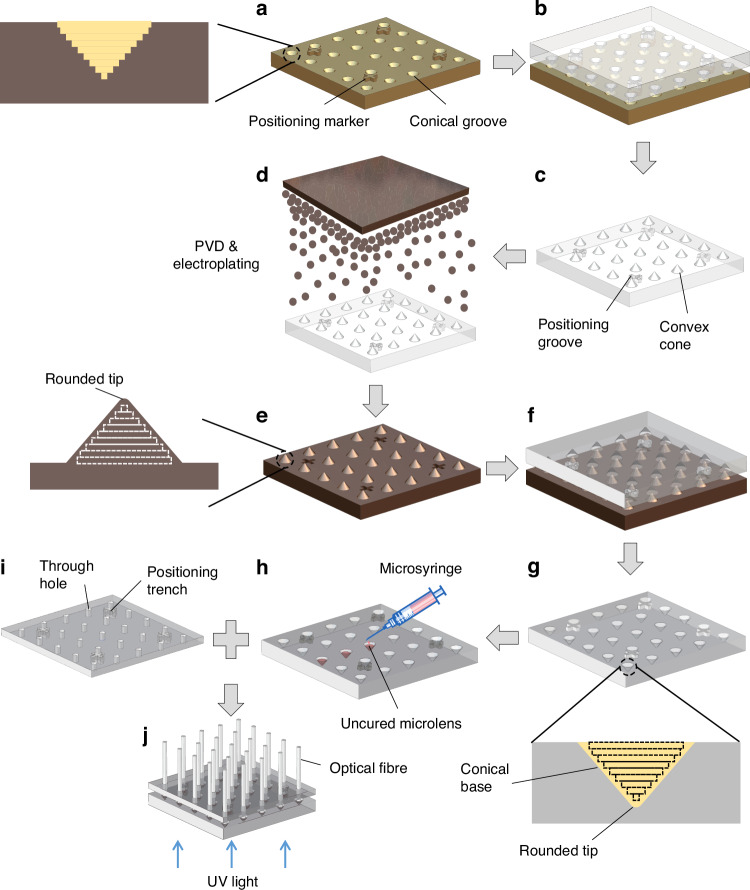
Fabrication process flow of conical microlens optical fibres. **a** A 3D-printed template with an array of conical grooves and 4 protruded ‘+‘ alignment markers at the corners. The enlarged view shows that the conical surface of each groove has a layered texture and is not smooth. **b** Polydimethylsiloxane (PDMS) is used to transfer patterns. **c** The first PDMS mould. **d** Physical vapour deposition (PVD) and electroplating. **e** Cu-coated mould. The inset shows that the layered texture is smoothened and the tip of the cone is rounded. **f** PDMS is used to transfer patterns again. **g** The second PDMS mould with conical grooves. **h** The same volume (~0.15 μL) of NOA81 liquid is deposited into each conical groove. **i** A 3D-printed optical fibre buncher with many through holes. **j** UV light is used to cure the conical microlenses on top of the optical fibres

Here, we present a pioneering method for fabricating a conical microlens on an optical fibre. First, a template with a conical groove array was designed with 3D CAD (Computer Aided Design) software (Fig. 9a). The size of the conical groove is equal to the abovementioned conical microlens, and four ‘+‘ markers are positioned out of plane at the four corners. Then, this template was fabricated using an ultrahigh precision 3D printing method (microArch® S140, BMF Precision Tech Inc.). Next, polydimethylsiloxane (PDMS) is used to transfer patterns of the template (Fig. 9b). This PDMS mould has convex cones and positioning grooves (Fig. 9c).

To smooth the rough surface of the convex cones due to the layered texture caused by the 3D printing method and to round the sharp tip of the convex cones, the PDMS mould is electroplated. A several nanometre thick Cu layer was coated on the PDMS surface using the physical vapour deposition method (PVD). The coated PDMS was then electroplated with Cu for 7 h at 1 A/dm^2^ electric current density (Fig. 9d). Since Cu has a faster deposition rate at positions with higher current density, the edges of the layered texture are connected, forming a smooth surface, and the sharp tip of each convex cone is rounded (Fig. 9e).

Subsequently, the Cu-coated PDMS mould was transferred to another PDMS mould (Fig. 9f), and this second PDMS mould has conical grooves and ‘+‘ positioning markers (Fig. 9g).

The material used for the microlens is NOA81, which is liquid and UV curable. A microsyringe was used to deposit the same volume of NOA81 (0.15 μL/drop) into each conical groove (Fig. 9h).

To mount each microlens on each optical fibre end in a batch process, an optical fibre buncher was designed and fabricated by the 3D printing (Fig. 9i). The optical fibre buncher has 4 ‘+‘ positioning trenches and an array of through-holes, each corresponding to the position of a conical groove in the second PDMS mould. The through-holes have a diameter of 0.28 mm, which is slightly larger than the diameter of the plastic optical fibre (0.25 mm) to address potential fabrication errors with the 3D printing method (±0.025 mm).

Thereafter, the optical fibre buncher was mounted on the second PDMS mould by carefully aligning the positioning trenches of the former to the protruded positioning markers of the latter under an optical microscope. As a result, each through-hole in the optical fibre buncher is well aligned to one conical groove in the second PDMS mould. Then, the optical fibres were manually threaded into the through-holes to contact the NOA81 microlenses (Fig. 9j), followed by UV illumination to cure the NOA81. With this approach, each conical microlens was firmly fixed on the end of the corresponding optical fibre.

Oxygen inhibits the free-radical polymerisation of liquid NOA81, and the permeability of PDMS in air ensures that an ultrathin surface layer of NOA81 remains uncured near each PDMS surface, though most of the body part of NOA81 is already hardened^37,38^. This uncured layer facilitates the easy detachment of the NOA81 microlenses from the second PDMS mould. Finally, many conical microlens optical fibres ( ~ 200 pieces) are obtained with this batch process. Interestingly, the moulds and the fibre buncher can be reused for more fabrication runs of conical microlens optical fibres.

In the future, we aim to simplify this manual process through automation. There are two possible approaches to achieve this automation:

1. AI-assisted robots: Artificial intelligence (AI) can be utilised to identify the through-holes in both the dome and the buncher. Subsequently, AI can control a robotic arm to insert the plastic microlensed fibres into these holes. The integration of AI with industrial processes has become increasingly popular in recent years due to the rapid advancements in AI technology, and it has the potential to significantly enhance automated fabrication.
2. Liquid waveguides: Traditional plastic optical fibres could be replaced with new liquid optical guides. Although a recent study attempted to use liquid optical guides by filling silicone elastomer into hollow pipelines within a 3D-printed black substrate^16^, the waveguiding effect was not well achieved due to the layered texture and black colour of the 3D-printed pipeline inner surfaces. Moreover, that recent study did not address the optical design criteria essential for optical waveguides in ACEs. In the future, we can develop microlensed liquid optical guides, consisting of a microlens, liquid optical guide core, and cladding, based on the design criteria discussed in this article. Further, these microlensed liquid optical fibres may be incorporated into the ACEcam components through spin coating.

#### Fabrication of the PDMS mould

1. A transparent elastomeric PDMS material and a curing agent are mixed with a mass ratio of 10:1.
2. The mixture is stirred thoroughly.
3. The mixture is centrifuged for 2 min at a speed of 1400 rpm to remove bubbles.
4. The mixture is poured on the surface of the conical groove template prefabricated by the ultrahigh precision 3D printing method.
5. The PDMS layer on the template is placed into a vacuum pump under a vacuum environment for 3 h.
6. The PDMS layer on the template is annealed at 85^o^C for 45 min.
7. The cured PDMS layer is peeled off from the template, forming the PDMS mould.

#### Fabrication of liquid microlenses

1. A microsyringe (volume 0.15 μL/drop) is used to hold the NOA81 liquid.
2. The microsyringe is used to inject the same volume of NOA81 into each conical groove.

#### Mounting the microlenses on the optical fibre

1. The ‘+‘ positioning trenches in the optical fibre buncher are precisely aligned with the protruded ‘+‘ positioning markers in the second PDMS mould under an optical microscope.
2. The optical fibres are individually inserted into the through-holes in the optical fibre buncher until they contact the conical grooves filled with liquid NOA81.
3. The liquid NOA81 is cured by UV illumination for 2 min.
4. The optical fibre buncher is carefully removed from the other end of the optical fibres.
5. The conical microlens optical fibres are removed from the second PDMS mould and further UV cured.

#### Alignment error analysis

The alignment markers on the PDMS mould and positioning trenches in the optical fibre buncher are designed to be the same size to ensure precise alignment. During the alignment process, the elasticity of the PDMS allows the alignment markers on the PDMS to fit into the positioning trenches in the optical fibre buncher, despite being the same size. This alignment process mimics the hard contact alignment used in common multiple photolithography, ensuring high positioning accuracy across different layers. Therefore, the alignment accuracy is correspondingly high.

The alignment error primarily arises from the fabrication error of these markers, which is less than 0.025 mm (microArch® S140, BMF Precision Tech Inc.). This fabrication error can be further reduced by using devices with a higher precision.

Based on our optical path analysis of this alignment error, when the optical axis of the optical fibre and microlens are slightly deviated (~10 µm), the relationship between the upper limit angle and the half-apex angle (Supplementary Fig. S11a), and the lower limit angle and the half-apex angle (Supplementary Fig. S11b) still comply with the principles described in Materials and methods: Acceptance angle of the plastic optical fibre capped with a conical microlens. Thus, this slight error can be considered negligible. Only when the deviation becomes too large (Supplementary Fig. S11c), the acceptance angle of the optical fibre deviates significantly, which would affect the detection performance. Therefore, utilising four markers to ensure a hard contact alignment is crucial for maintaining optimal performance.

### Calculation of maximum angular velocities

#### Using a CMOS chip as the photodetection unit

In our testing setup (Fig. 4c), the moving object is mimicked by 5 equally spaced and sequentially driven LEDs. The delay time Δ*t*_1_ when one object moves from one side of the dome to the opposite side can be determined by the FOV and the angular speed *ω* as follows:

36 $$\varDelta {t}_{1}=\frac{{\rm{FOV}}}{\omega }$$

The moving object is mimicked by 5 sequentially driven LEDs; thus, Δ*t*_1_ should be greater than or equal to the response time Δ*t*_*dec*_ of the photodetector,

37 $$\varDelta {t}_{1}\ge \varDelta {t}_{dec}$$

The combination of both equations gives

38 $$\omega \le \frac{{\rm{FOV}}}{\varDelta {t}_{dec}}$$

and, thus, the maximum angular velocity is given by

39 $${\omega }_{\max }=\frac{{\rm{FOV}}}{\varDelta {t}_{dec}}$$

The response time of a CMOS chip with a frame rate of 30 Hz is 33.3 ms. Correspondingly, the highest angular perception speed is

40 $${\omega }_{\max }=\frac{180^{\circ} }{33.3\times {10}^{-3}}=5.4\times {10}^{3}\,{\rm{deg }}/{\rm{s}}$$

#### Using a photodiode array as the photodetection unit

When a photoiode array with 5 electromagnetically shielded photodiodes (ElecFans) is used for photodetection, the response time is 31.9 μs (equivalently, 31.3 kHz). In this case, the highest angular perception speed is

41 $${\omega }_{\max }=\frac{{\rm{FOV}}}{\varDelta {t}_{dec}}=\frac{180^{\circ} }{31.9\times {10}^{-6}}=5.6\times {10}^{6}\,{\rm{deg }}/{\rm{s}}$$

## Supplementary information

Supplementary information

Supplementary video 1

Supplementary video 2

Supplementary video 3

Supplementary video 4

## Data Availability

The data that supports the plots within this paper and other findings of this study are available from the corresponding authors upon reasonable request.

## Code availability

The codes that support the findings of this study are available from the corresponding authors upon reasonable request.
