# Supplementary material for: Optical fibre based artificial compound eyes for direct static imaging and ultrafast motion detection
Source: Light Sci Appl. 2024 Sep 18;13:256. doi: 10.1038/s41377-024-01580-5 (PMC11410978; doi:10.1038/s41377-024-01580-5)
Supplement: Supplementary file 1 — Supplementary information [file 41377_2024_1580_MOESM1_ESM.pdf]

Supplementary Information for Optical Fibre based Artificial Compound Eyes for Direct Static Imaging and Ultrafast Motion Detection

Heng Jiang[1,2], Chi Chung Tsoi[1,2], Weixing Yu[3], Mengchao Ma[4], Mingjie Li[1], Zuankai Wang[5], Xuming Zhang[1,2,6]*

[1]Department of Applied Physics, The Hong Kong Polytechnic University, Hong Kong 999077, China
[2]Photonics Research Institute, The Hong Kong Polytechnic University, Hong Kong 999077, China
[3]Key Laboratory of Spectral Imaging Technology, Xi'an Institute of Optics and Precision Mechanics, Chinese Academy of Sciences 710119, China
[4]Anhui Province Key Laboratory of Measuring Theory and Precision Instrument, School of Instrument Science and Opto-Electronics Engineering, Hefei University of Technology, Hefei 230009, China
[5]Department of Mechanical Engineering, The Hong Kong Polytechnic University, Hong Kong 999077, China
[6]Research Institute for Advanced Manufacturing, The Hong Kong Polytechnic University, Hong Kong 999077, China
***Correspondence:** Xuming Zhang (xuming.zhang@polyu.edu.hk)

Table of contents:

Supplementary Information 1: Supplementary figures 2
Supplementary Information 2: The critical parameter *m* of a camera 11
Supplementary Information 3: Principle of the Lucas-Kanade method 15
Supplementary Videos 18

Supplementary Information 1: Supplementary figures

Table S1 | Detailed comparison of this work with reported artificial compound eyes (ACEs) and natural compound eyes (NCEs)

Specification	BIC	-	-	Curv -ACE	Digital cameras	BAC -eye	NCEs	ACEcam
Field of view	90°	140°	-	$180^o \times 60^o$	160°	170°	150°-180°	180°
Crosstalk prevention	×	×	√	√	√	√	√	√
Nearly infinite depth of field	√	√	√	√	√	×	√	√
Distance estimation	×	×	×	×	×	×	√	√
Optical guiding	×	×	√	×	×	√	√	√
Panoramic Imaging	×	×	×	×	√	√	√	√
Real-time direct imaging	×	×	×	×	×	√	√	√
Dynamic detection	×	×	×	√	×	×	√	√
Fast motion detection	×	×	×	×	×	×	√	Ultrahigh
Reference in the main text	[14]	[18]	[10]	[11]	[12]	[16]	[1,3,4,7,16 ,26]	-
Literature source*	AOM	AFM	Science	PNAS	Nature	NC	-	This work
Generation	1st generation ACEs		2nd generation ACEs				-	-

*: AOM: Advanced Optical Materials; AFM: Advanced Functional Materials; PNAS: Proceedings of the National Academy of Sciences; NC: Nature Communications.

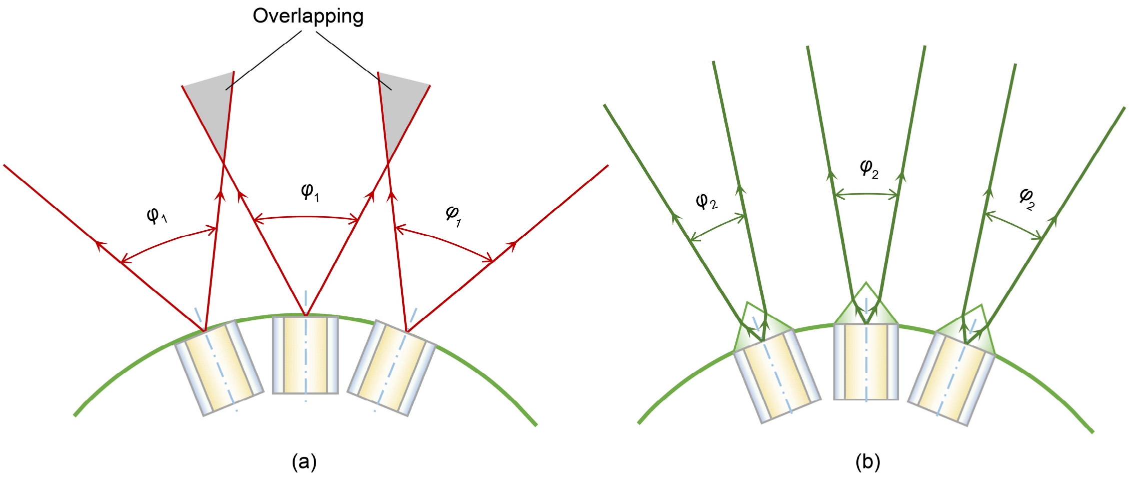

Figure S1 | Overlapping acceptance angles between adjacent optical fibres (i.e., ommatidia). a, Bare optical fibres have flat ends and a large acceptance angle (φ_1), which causes severe overlap. **b,** With conical microlenses, the acceptance angle (φ_2) can be narrowed to reduce this overlap.

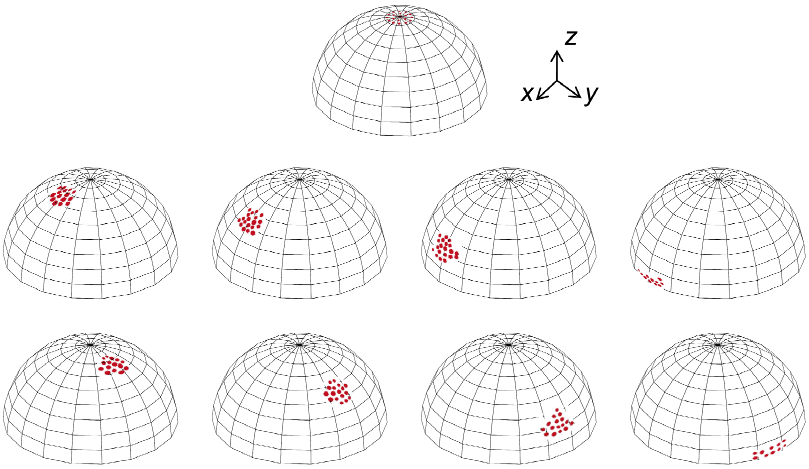

Figure S2 | Images of the laser spots projected onto the full-field camera. The images are acquired at 9 different incident angles (from 0° to 90° in the x direction and the y direction in steps of 22.5°). They are rendered on a hemispherical surface for easy visualization.

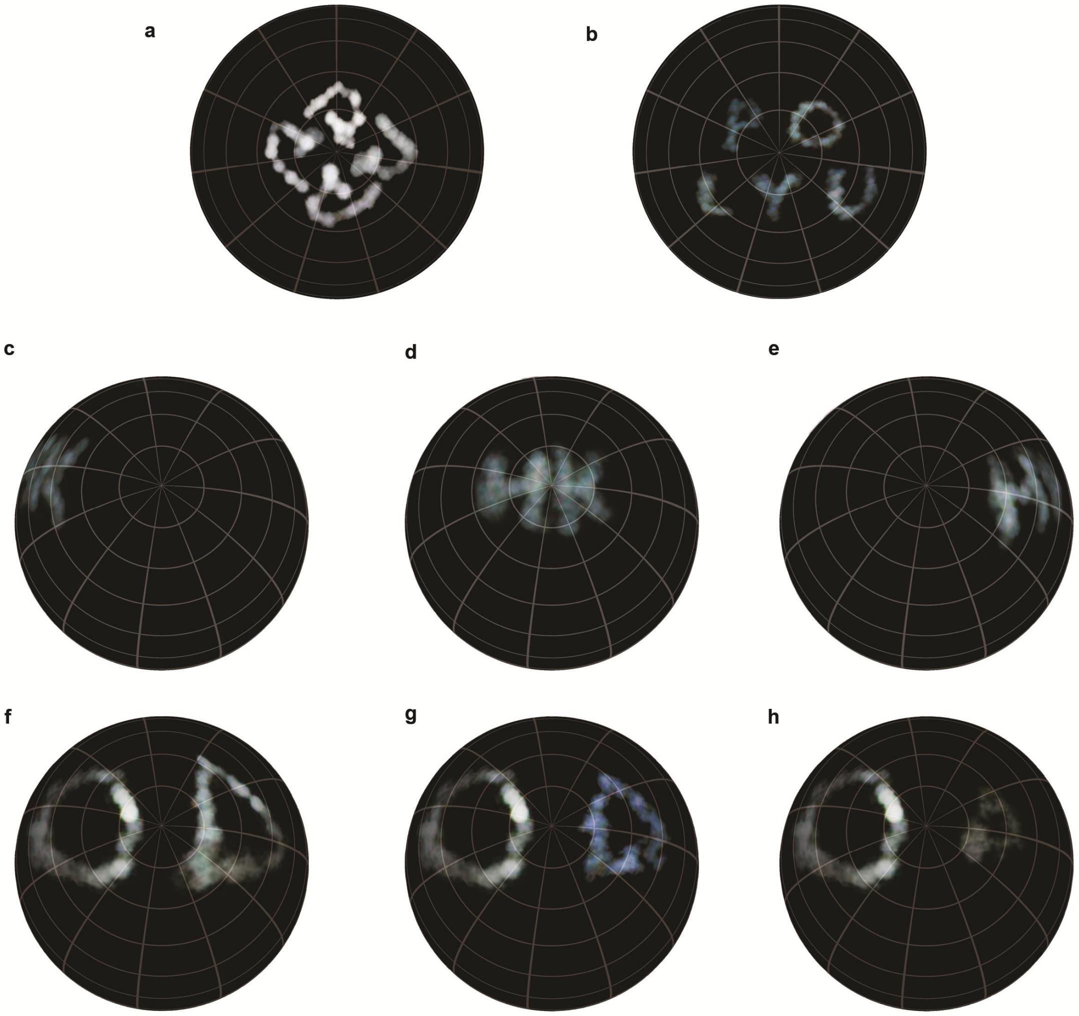

Figure S3 | Static imaging results of different object patterns captured by the ACEcam. **a,** Logo of The Hong Kong Polytechnic University. **b,** The letters 'POLYU', the abbreviation of The Hong Kong Polytechnic University. **c-e,** The letters 'HK' captured at three different polar angles relative to the centre of the camera: -50° (**c**), 0° (**d**), and 50° (**e**). **f-h,** Objects A (circle) and B (triangle) placed at angular positions of 40° and -40° with a fixed distance D_A and a varying distance D_B (from 2, 8 to 14 mm), respectively.

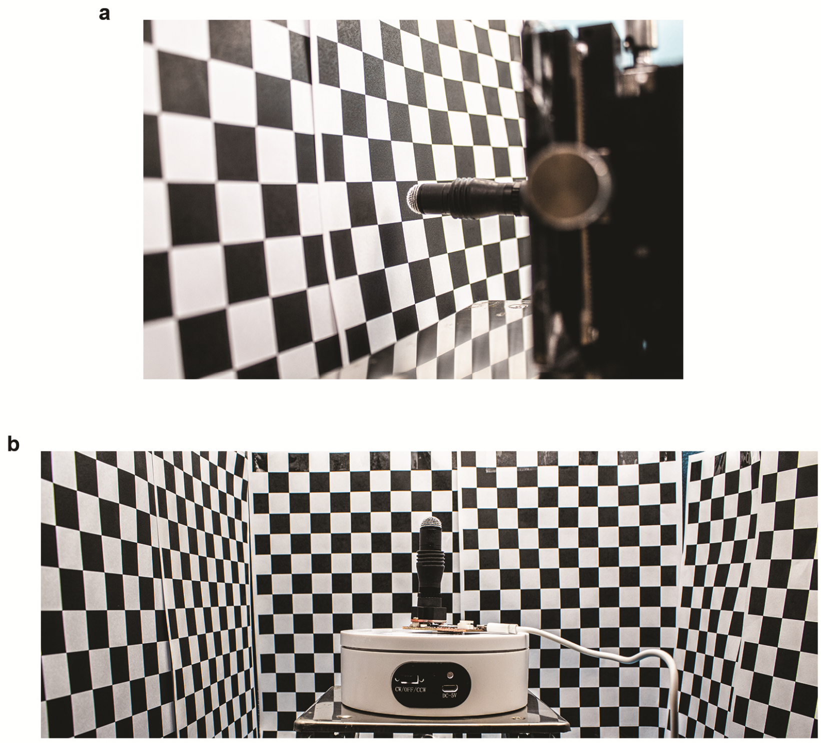

Figure S4 | Setups for optical flow detection. a, The ACEcam is positioned 10 mm in front of a checkerboard pattern and translated. **b,** The ACEcam is rotated on a rotary stage.

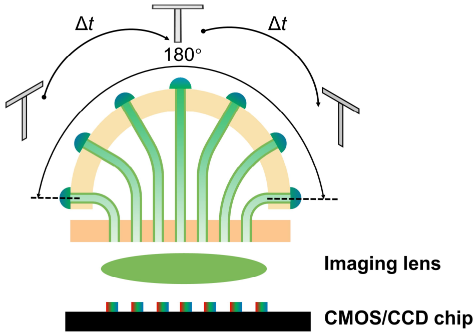

Figure S5 | Experimental setup to generate high angular velocities for the dynamic response measurement. Three objects 'T' are evenly spaced along 180° and lit up successively with a delay time Δt, and a CMOS chip is employed to record the patterns.

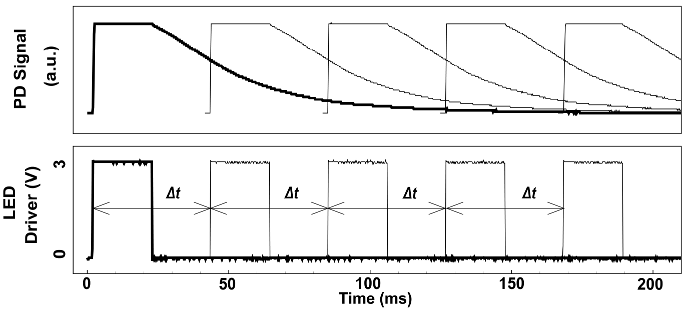

Figure S6 | Dynamic angular perception results when $f_{flicker}$ = 24 Hz. The lower half shows the input signal to the LEDs, and the upper half shows the signals recorded by the five photodiodes.

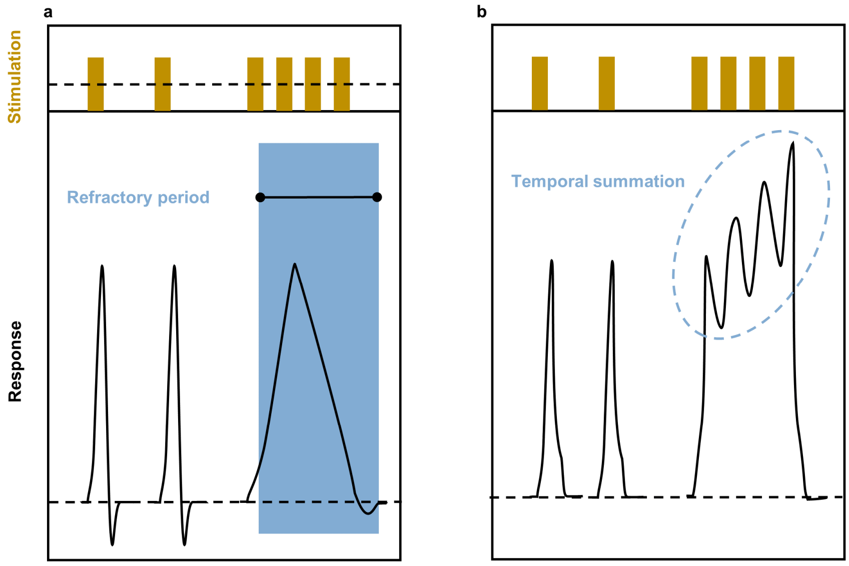

Figure S7 | Response characteristics of spiking neurons (a) and nonspiking graded neurons (b). a, In spiking neurons, an action potential can be triggered by strong stimulation. Then, the neuron enters a refractory period, during which it does not respond to stimulation. **b,** In nonspiking graded neurons, sequential stimulation leads to nonlinear temporal summed responses.

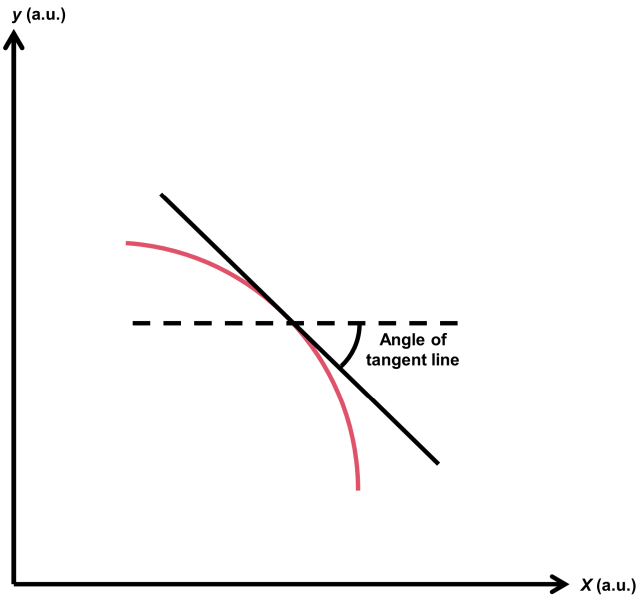

Figure S8 | Schematic diagram of the angle of the tangent line. The Cartesian coordinate system, featuring the *x*-*y* axis, defines the 2D plane. The black solid line is the tangent line of the curved surface (red line) of a microlens. The angle of the tangent line is determined with respect to the horizontal line (dotted line).

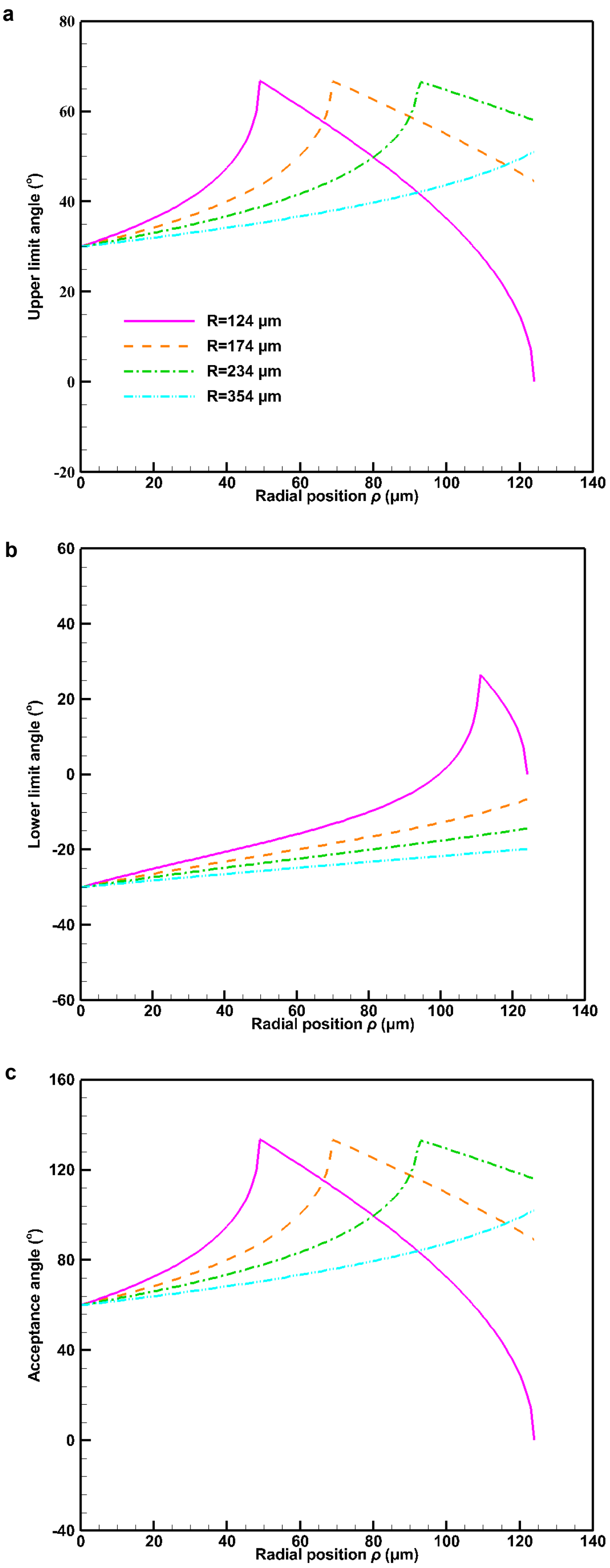

Figure S9 | Acceptance angles of the *spherical* microlens optical fibres for the microlenses with different radii. a, The upper limit angle, which is theoretically calculated, versus the radial position ρ at which the light hits the microlens surface (Fig. 6c in the main

text). **b,** The lower limit angle versus ρ (Fig. 6d in the main text). **c**, The acceptance angle versus ρ.

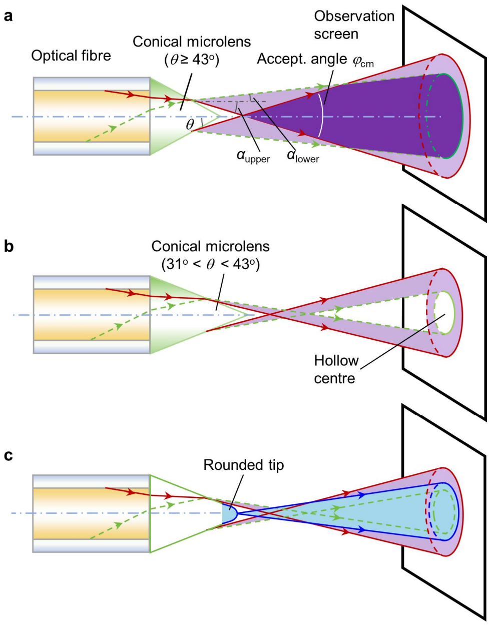

Figure S10 | Divergence angles of the optical fibres capped with *conical microlenses* with different half-apex angles. Red paths represent light reflected from the upper core/cladding interface of the optical fibre, green paths represent light reflected from the lower core/cladding interface of the optical fibre, and blue paths represent light emitted from the rounded tip of the optical fibre**. a**, When the cone has a half-apex angle $\theta \geq 43^{o}$, the rays from the upper core/cladding interface go out at a *downward* angle*,* and those from the lower core/cladding interface go out at an *upward* angle, forming a solid circle on the observation screen. The acceptance angle is determined by the upper limit angle α_{upper}. **b**, When $31^{o} < \theta < 43^{o}$, the rays from the upper core/cladding interface and the lower core/cladding interface both travel *downwards*, causing a hollow central region on the observation screen. The rays from the hollow central region cannot be collected by the conical microlens optical fibre. **c**, The tip of the cone is rounded to prevent the appearance of the hollow central region.

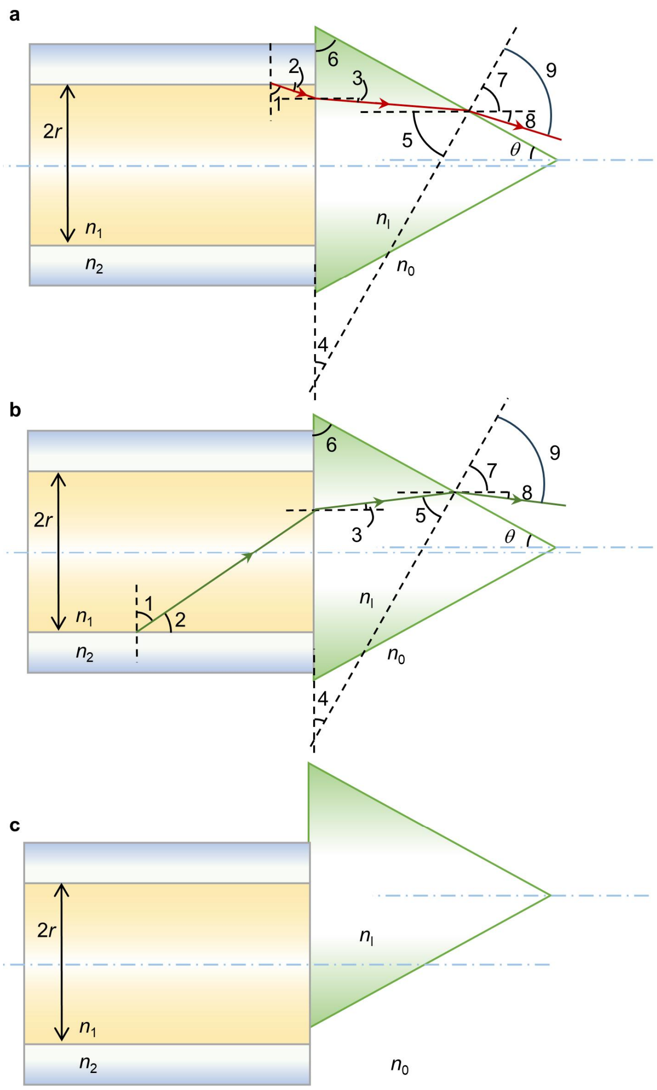

Figure S11 | Light paths in deviated conical microlens optical fibres for the alignment error analysis. a-b, In the optical fibre capped with a slightly deviated conical microlens, the light has various paths. The light experiences no reflection in the conical surface after being reflected from the upper (**a**) or lower (**b**) core/cladding interface. **c,** Then conical microlens deviates significantly from the optical fibre.

Supplementary Information 2: The critical parameter *m* of a camera

The focusing property of the imaging lens used in ACEcam can be evaluated using geometrical optics. As shown in Fig. S12, the point p is the image position of the object point P. However, if p is not in the focal plane, a defocused, blurred circle appears in the detector plane, where p' denotes the centre of the blurred circle. Based on the geometrical analysis, the relationship among the blurred circle's diameter d_2, the lens diameter D, the distance from the lens to the focal plane s, the focal length f, and the object distance u can be expressed as

$$d_2 = Ds\left(\frac{1}{f} - \frac{1}{u} - \frac{1}{s}\right). \quad \text{(S1)}$$

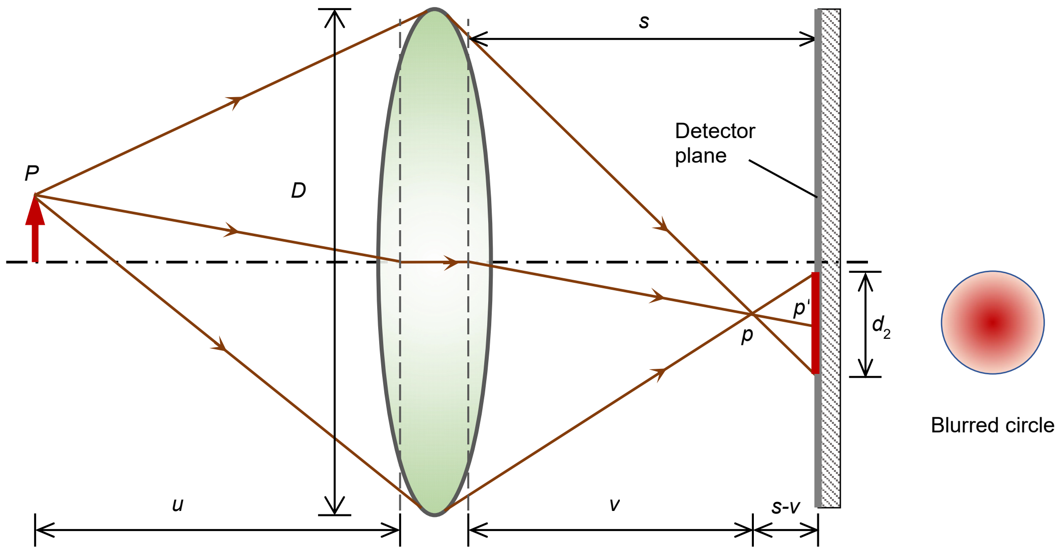

Figure S12 | Imaging property of a lens using geometrical optics. Here, p is the image point of the object point P, p' is the centre point of the blurred circle in the detector plane, and d_2 is the diameter of the blurred circle. The variables D, u, v and s denote the lens diameter, object distance, image distance, and distance between the lens and detector plane, respectively.

Due to the diffraction and the nonideal properties of lenses[1-2], the intensity distribution $h(x, y)$ of the blurred circle follows a two-dimensional Gaussian distribution,

$$h(x,y) = \frac{1}{2\pi\sigma^2} e^{-\frac{x^2+y^2}{2\sigma^2}}, \qquad \text{(S2)}$$

where σ is the spread parameter, which has a linear relationship with d_2 (k is a constant):

$$\sigma = kd_2, \ \text{here } k > 0. \qquad \text{(S3)}$$

In fact, $h(x, y)$ is the point spread function of this camera, as discussed below. We can combine Eqs. (S1) and (S3) to formulate the relationship between the spread parameter σ and the object distance u:

$$\sigma = kDs(\frac{1}{f} - \frac{1}{u} - \frac{1}{s}). \qquad \text{(S4)}$$

For the camera used in our setup, the variables k, D, s and f are fixed camera parameters. Thus, Eq. (S4) can be simplified as

$$\sigma = mu^{-1} + c, \qquad \text{(S5)}$$

where m and c are constants.

To determine the values of m and c using linear fitting, the values of σ and u under several different conditions should be measured. We first consider how to measure σ.

We introduce a step edge function along the y direction in the image plane $f(x, y)$, which can be formulated as

$$f(x,y) = a + bu(x), \qquad \text{(S6)}$$

where a is the initial intensity, b is the height of the step, and $u(x)$ is the standard unit step function. The observed image $g(x, y)$ is then the convolution of $f(x, y)$ and the point spread

function $h(x, y)$:

$$g(x, y) = f(x, y) * h(x, y), \tag{S7}$$

where * represents the convolution operation. The derivative of g along the gradient direction can be written as

$$\frac{\partial g}{\partial x} = h(x, y) * \frac{\partial f}{\partial x} = h(x, y) * b\delta(x), \tag{S8}$$

where $\delta(x)$ is the derivative of $u(x)$ along the x direction, which has the form of a Dirac delta function. This expression can be written as

$$\begin{aligned}\frac{\partial g}{\partial x} &= \int_{-\infty}^{\infty}\int_{-\infty}^{\infty} h(\varepsilon, \eta) b\delta(x-\varepsilon) d\varepsilon d\eta \\ &= \int_{-\infty}^{\infty}[\int_{-\infty}^{\infty} h(\varepsilon, \eta) b\delta(x-\varepsilon) d\varepsilon] d\eta = b\int_{-\infty}^{\infty} h(x, \eta) d\eta\end{aligned}. \tag{S9}$$

$\theta_2(x)$, which is also called the line spread function, can be used to represent this line integral:

$$\theta_2(x) = \int_{-\infty}^{\infty} h(x, y) dy. \tag{S10}$$

Then, Eq. (S9) can be written as

$$\frac{\partial g}{\partial x} = b\theta_2. \tag{S11}$$

The integration of this equation along the x direction is

$$\int_{-\infty}^{\infty} \frac{\partial g}{\partial x} dx = \int_{-\infty}^{\infty} b\theta_2 dx = b\int_{-\infty}^{\infty}\int_{-\infty}^{\infty} h(x, y) dx dy. \tag{S12}$$

The integral of the point spread function should be unity, i.e.,

$$\int_{-\infty}^{\infty}\int_{-\infty}^{\infty} h(x, y) dx dy = 1. \tag{S13}$$

Therefore, we have

$$\int_{-\infty}^{\infty} \frac{\partial g}{\partial x} dx = b . \quad \text{(S14)}$$

Thus, $\theta_2(x)$ can be expressed as

$$\theta_2(x) = \frac{\frac{\partial g}{\partial x}}{\int_{-\infty}^{\infty} \frac{\partial g}{\partial x} dx} . \quad \text{(S15)}$$

Since the point spread parameter σ of a line spread function[3] is the standard deviation of the line spread function $\theta_2(x)$, we have

$$\sigma = \sqrt{\int_{-\infty}^{\infty} (x - \bar{x})^2 \theta_2(x) dx} , \quad \text{(S16)}$$

where $\bar{x}$ represents the average of the line spread function. Thus, it follows that

$$\bar{x} = \int_{-\infty}^{\infty} x \theta_2(x) dx . \quad \text{(S17)}$$

If the greyscale gradient $\frac{\partial g}{\partial x}$ at the edge of an observed image is obtained, the line spread function $\theta_2(x)$ can be determined. Then, the average $\bar{x}$ and the point spread parameter σ can be obtained. Therefore, the point spread parameter σ indirectly represents the gradient at the edge. If several values of the point spread parameter σ and the object distance u are given, the values of m and c can be determined using Eq. (S5). More importantly, m is the slope of the relationship between σ and u^{-1} and thus represents the extent to which a camera's imaging quality (characterized by the gradient at the edge) is affected by a change in the object distance. In this work, this parameter m is defined innovatively as the *critical parameter*.

Supplementary Information 3: Principle of the Lucas-Kanade method

In this work, we assume that the grey invariant hypothesis is valid, that is, that a pixel's grey value at a spatial site remains fixed in each image[4-5].

The pixel at (x, y) at time t changes its position to $(x+dx, y+dy)$ at time $(t+dt)$. Based on our initial assumption, the grey value I should follow

$$I(x+dx, y+dy, t+dt) = I(x, y, t)\,. \tag{S18}$$

When the left side of the above equation is Taylor expanded, we obtain

$$I(x+dx, y+dy, t+dt) \approx I(x, y, t) + \frac{\partial I}{\partial x}dx + \frac{\partial I}{\partial y}dy + \frac{\partial I}{\partial t}dt\,. \tag{S19}$$

The combination of Eqs. (S18) and (S19) yields

$$\frac{\partial I}{\partial x}dx + \frac{\partial I}{\partial y}dy + \frac{\partial I}{\partial t}dt = 0\,. \tag{S20}$$

The above expression can be rewritten as

$$\frac{\partial I}{\partial x}dx + \frac{\partial I}{\partial y}dy = -\frac{\partial I}{\partial t}dt\,. \tag{S21}$$

Here, we use some variables to simplify the equation,

$$\frac{dx}{dt} = u_2,\ \frac{dy}{dt} = v_2,\ \frac{\partial I}{\partial x} = I_x,\ \frac{\partial I}{\partial y} = I_y\,. \tag{S22}$$

Then, the equation can be written in matrix form as follows:

$$\begin{bmatrix} I_x & I_y \end{bmatrix} \begin{bmatrix} u_2 \\ v_2 \end{bmatrix} = -I_t\,. \tag{S23}$$

As there are two variants, u_2 and v_2, a single point is insufficient for calculating their values. To address this issue, a calculation pixel window with a size of $w \times w$ is established. To calculate the variants, we assume that the pixels in the same window follow the same

motion. Consequently, each window contains w^2 pixels, resulting in w^2 functions. The equation can then be expressed in matrix form as follows:

$$\begin{bmatrix} I_x & I_y \end{bmatrix}_k \begin{bmatrix} u_2 \\ v_2 \end{bmatrix} = -I_{tk}, \text{ here } k = 1, \ldots, w^2. \tag{S24}$$

Let

$$A = \begin{bmatrix} \begin{bmatrix} I_x & I_y \end{bmatrix}_1 \\ \cdot \\ \cdot \\ \cdot \\ \begin{bmatrix} I_x & I_y \end{bmatrix}_{\omega^2} \end{bmatrix}, \; w = \begin{bmatrix} u_2 \\ v_2 \end{bmatrix}, \; b_2 = \begin{bmatrix} I_{t1} \\ \cdot \\ \cdot \\ \cdot \\ I_{t\omega^2} \end{bmatrix}. \tag{S25}$$

Then, the above equation becomes

$$Aw = -b_2. \tag{S26}$$

This is an overdetermined linear equation, and the least square method is adopted as follows:

$$\widehat{w} = -\begin{bmatrix} A^T & A \end{bmatrix}^{-1} A^T b_2. \tag{S27}$$

With this approach, the optical flow u_2 and v_2 in a window can be computed, and after calculating the values for all windows in an image, the overall optical flow can finally be obtained.

References

1. Horn, B., Klaus, B. & Horn, P. *Robot vision*. (MIT Press, 1986).
2. Pentland, A. P. A new sense for depth of field. *IEEE Transactions on Pattern Analysis and Machine Intelligence*, 523-531 (1987).
3. Subbarao, M. & Gurumoorthy, N. Depth recovery from blurred edges. Proceedings CVPR'88: The Computer Society Conference on Computer Vision and Pattern Recognition. Ann Arbor, MI, USA: IEEE, 1988, 498-503.
4. Lucas, B. D. & Kanade, T. An iterative image registration technique with an application to stereo vision. Proceedings of the 7th International Joint Conference on Artificial intelligence. Vancouver, BC, Canada: Morgan Kaufmann Publishers Inc., 1981, 674-679.
5. Fleet, D. J. & Langley, K. Recursive filters for optical flow. *IEEE Transactions on Pattern Analysis and Machine Intelligence* **17**, 61-67 (1995).

Supplementary Videos

Supplementary Video 1: ACEcam detects two objects that are placed at angular positions of 40° and -40° with a fixed distance D_A or a varying distance D_B. When the distances of both objects are the same, the image sizes are identical. As D_B increases, the size of object B decreases (00:03 - 00:18); in contrast, as D_B decreases, the size of object B increases (00:22 - 00:33). Although the image size of the object varies with the change in distance, the focus is always retained.

Supplementary Video 2: Dynamic angular perception experiment using a conventional CMOS imaging chip as the photodetector. Five sequentially illuminated LEDs are evenly arranged over 180° to mimic an object with high angular velocity. The CMOS chip has a frame rate of 30 Hz, corresponding to an angular velocity of 5.4×10^3 deg s^{-1}.

Supplementary Video 3: Dynamic angular perception experiment on actual objects. Three objects 'T' are evenly arranged over 180° to mimic an object with high angular velocity. The CMOS chip is employed for object recording; however, at high flicker frequencies, there is a tendency for certain objects to be missed during recording.

Supplementary Video 4: Dynamic angular perception experiments using a photodiode array that consists of 5 electromagnetically shielded photodiodes. The light emitted by flicker LEDs (representing a moving object) is recorded by the photodiodes. The number

beneath each sub-video corresponds to the depicted photodiode in the right corner, recording the signal emitted by the corresponding LED. The highest detection frequency is 31.3 kHz, corresponding to an angular velocity of 5.6×10^{6} deg s^{-1}.
